# Local conditions matter: Minimal and variable effects of soil disturbance on microbial communities and functions in European vineyards

**DOI:** 10.1371/journal.pone.0280516

**Published:** 2023-01-27

**Authors:** Magdalena Steiner, Martin Pingel, Laurent Falquet, Brice Giffard, Michaela Griesser, Ilona Leyer, Cristina Preda, Deniz Uzman, Sven Bacher, Annette Reineke

**Affiliations:** 1 Ecology and Evolution, Department of Biology, University of Fribourg, Fribourg, Switzerland; 2 Department of Applied Ecology, Geisenheim University, Geisenheim, Germany; 3 Swiss Institute of Bioinformatics, Fribourg, Switzerland; 4 Bordeaux Sciences Agro, UMR 1065 SAVE Santé et Agroécologie du Vignoble, INRA, ISVV, Gradignan, France; 5 Department of Crop Sciences, Institute of Viticulture and Pomology, University of Natural Resources and Life Sciences Vienna (BOKU), Tulln, Austria; 6 Department of Natural Sciences, Aleea Universitatii, Ovidius University of Constanta, Constanta, Romania; 7 Department of Crop Protection, Geisenheim University, Geisenheim, Germany; Carnegie Mellon University, UNITED STATES

## Abstract

Soil tillage or herbicide applications are commonly used in agriculture for weed control. These measures may also represent a disturbance for soil microbial communities and their functions. However, the generality of response patterns of microbial communities and functions to disturbance have rarely been studied at large geographical scales. We investigated how a soil disturbance gradient (low, intermediate, high), realized by either tillage or herbicide application, affects diversity and composition of soil bacterial and fungal communities as well as soil functions in vineyards across five European countries. Microbial alpha-diversity metrics responded to soil disturbance sporadically, but inconsistently across countries. Increasing soil disturbance changed soil microbial community composition at the European level. However, the effects of soil disturbance on the variation of microbial communities were smaller compared to the effects of location and soil covariates. Microbial respiration was consistently impaired by soil disturbance, while effects on decomposition of organic substrates were inconsistent and showed positive and negative responses depending on the respective country. Therefore, we conclude that it is difficult to extrapolate results from one locality to others because microbial communities and environmental conditions vary strongly over larger geographical scales.

## Introduction

There is growing evidence that diverse bacterial and fungal communities sustain multifunctionality of soils [1–3]. Agricultural soil management should therefore aim to conserve and promote diverse microbial communities [4]. Soil microbial communities are affected by many factors such as geography [5–7], habitat type [2], soil parameters [8, 9], and agricultural management [10, 11]. Agricultural soil management includes practices with the purpose of removing weeds, namely tillage or herbicide application. These weed control practices pose a disturbance to the soil system either by direct mechanical disruption (in case of tillage) or by introduction of chemical compounds (in case of herbicide). Both practices have in common that removal of aboveground vegetation reduces inputs of plant biomass to the soil leading to long term depletion of soil organic carbon [12, 13].

With regard to conserving biodiversity, soil disturbances due to weed control practices are generally associated with an impoverished diversity of macrobiota such as plants or soil arthropods [14, 15]. For soil-inhabiting microbes, however, evidence regarding the relationship between disturbance and diversity is mixed [15]. In some studies, weed control resulted in a decrease of bacterial and fungal diversity, while other studies reported an increase of bacterial diversity after weed control [16–19]. Thus, soil disturbance by tillage and herbicide application may not lead to a general loss of species across all microbial groups and respective effects might rather depend on other environmental conditions and stressors [16, 20–24]. To the best of our knowledge, a thorough understanding of the consistency of disturbance effects of weed control practices on soil microbial diversity is lacking.

Soil functions associated with microbial activities also show varying responses to weed control practices. Microbial respiration and decomposition represent functions crucial for nutrient cycling in the soil and are indispensable for sustainable soil functioning and agriculture [25]. Microbial respiration, considered as a proxy for general microbial activity [26], has been found to increase shortly after tillage [27], but to decrease in a longer time span of about 40 days [28, 29]. Decomposition of organic material in soils represents a complex soil function composed of many steps carried out by different taxonomic groups, which may be affected by soil disturbance. Decomposition can increase after mechanical soil disturbance [12, 30], while knowledge on effects of chemical soil disturbance via herbicide application is scarce [31].

Grapevines as permanent crops are usually cultivated in rows providing the opportunity for extensive soil management, which means that inter-row vegetation is either not removed or a cover-crop is used to suppress weeds as an alternative to tillage or herbicide application [32]. Therefore, vineyard inter-rows may provide relatively little-disturbed soil habitats for microbial communities depending on the management strategy applied. Whether extensive soil management practices with reduced disturbances are beneficial or detrimental (or neutral) for microbial communities could be of critical importance for viticulture since the soil microbiome might indirectly affect fruit development and quality [33, 34].

Most studies focusing on the influence of soil disturbance on microbial communities were locally restricted to one or two experimental fields [35–38]. Because vine-growing regions vary greatly in terms of environmental conditions and viticultural management practices besides soil management, the local restrictions of previous studies and an unknown level of stochasticity limit the ability to draw general conclusions about the effects of disturbance on microbial diversity and their ecosystem functions. Additionally, biogeographic processes could have shaped soil microbial community patterns at the continental scale [6, 7, 39]. Ruling out the variability of site-specific conditions requires a large-scale experiment using widely harmonized disturbance gradients and standardized protocols, but this type of study is currently lacking for vineyards.

Here, we report on a large-scale field experiment to determine how soil disturbance associated with weed removal affected soil bacterial and fungal diversity and associated ecosystem functions (respiration, decomposition) in vineyards across five European countries. Three levels of soil disturbance (either tillage or herbicide application), representing an intensity gradient from low to high disturbance, were applied within each country. Our objectives were (1) to test if there are consistent effects of disturbance on microbial alpha-diversity and community composition; (2) to test if soil functions in vineyards are consistently affected by increased soil disturbance; and (3) to assess the magnitude and transferability of these effects in light of varying environmental conditions.

## Materials and methods

### Study sites & disturbance gradient

The study was carried out in vine-growing regions located in five European countries (Fig S1 in S1 File): Austria (AT, regions *Kamptal*, *Kremstal*, *Leithaberg*), Switzerland (CH, *Valais*), Germany (DE, *Rheinhessen*), France (FR, *Bordeaux Libournais*), and Romania (RO, *Dobrogea*). All vineyards selected for the study exhibited a cane-trained or spur-trained growing system with non-cropped areas between grapevine rows (hereinafter referred as inter-rows). Before the start of the study, inter-row soil management varied slightly between countries regarding several factors: duration of the vegetation cover, frequency, and inter-row rotation of soil management, which was carried out by tillage (Austria, Germany, France, and Romania) or herbicide application (Switzerland). Details on all vineyards are given in Table S1 in S1 File.

For all countries except Switzerland, 9 vineyards were selected. In each vineyard, 3 experimental plots (= 3 disturbance levels; see description below) with a minimum size of 4 inter-rows and 20 m row-length were prepared in 2015. In Switzerland, vineyards were small and situated in a mosaic-like landscape; therefore, an adapted study design was implemented, where each vineyard (n = 29) represented a plot with one disturbance level (low n = 10, intermediate n = 9, high n = 10).

Experimental plots were prepared according to three different types of inter-row management within each country. These inter-row management types represented a soil disturbance gradient of three levels (“low”, “intermediate”, “high”) within each country and thus were comparable across different European vine-growing regions. Low soil disturbance was characterized by permanent vegetation throughout the season in all inter-rows, which was cut several times a year. High soil disturbance was represented by complete removal of vegetation in all inter-rows either by tillage of the upper 10 cm soil layer 2–4 times during the growing season (in Austria, Germany, France, Romania) or by 1–4 herbicide applications per growing season starting in April until harvest (Switzerland). Intermediate disturbance was realized differently: either by alternating treatment, i.e., removal of vegetation in every second inter-row by tillage (Austria, Germany) or herbicide application (Switzerland), or by tillage followed by sowing of a green manure cover in every inter-row. The latter treatments were implemented either during winter (France) or summer (Romania). Although we aimed at harmonizing the disturbance treatment of experimental vineyards across all countries, we needed to allow for the above-described inter-country variation of the intermediate treatment. However, the intermediate treatment was always a disturbance level between the two extremes (no and complete vegetation removal) in all countries. Although this might affect the strength of results, we had to acknowledge that wine-growers have their personal experiences and preferences for soil management and need to attune to local conditions. A detailed description of the implementation of soil disturbance levels in each country is given in Table S2 in S1 File.

All vineyards were commercially managed, agrochemical products were applied according to the customs of the winegrowers and following the local guidelines for integrated viticulture except for three vineyards in Austria and one vineyard in France, which were organically managed (Table S1 in S1 File).

### Soil sampling, DNA extraction, sequencing, and processing

A brief overview of soil sampling and DNA extraction, sequencing and bioinformatic procedures is given here. For a detailed description, the reader is referred to Appendix 1 in S1 File.

In each country, soil samples were taken > 1 year after preparation of experimental plot (2016 and/or 2017) and at minimum 6 weeks after tillage/herbicide application around the time of grapevine flowering in early summer. Eight soil subsamples were taken in the two central inter-rows of each plot to a depth of 10 cm and were pooled afterwards into one mixed sample, summing up to a total of 405 soil samples (each 54 samples from Austria 2016, France 2017, Romania 2017, Germany 2016 and Germany 2017, and 77 samples from Switzerland 2016 and 58 from Switzerland 2017). In Switzerland, four samples were taken in intermediately disturbed plots (two per inter-row) in 2016, but only two samples (one per inter-row) were taken in 2017.

DNA was extracted from 0.25 g of mixed soil sample per inter-row by using the DNeasy PowerSoil Kit^®^ following the manufacturer’s protocol (QIAGEN N.V., Venlo, Netherlands). DNA sequencing was conducted for the bacterial V4 region of the 16S rDNA [40], and for the fungal internal transcribed spacer ITS2 [41, 42]. Sequencing of 291 bp (V4 region for bacteria) and 122 ~ 245 bp (ITS region for fungi) paired-end amplicons was conducted on a MiSeq Illumina machine at Génome Quebec Innovation Centre (Montreal, Canada).

For bacterial sequence analysis, the software package Mothur (version 1.39.5) was used [43] following the Standard Operating Procedure outlined on http://www.mothur.org/wiki/MiSeq_SOP. Sequence analysis for fungi was conducted using the software package PIPITS (version 1.3.x, [44]).

Data processing was done in R version 3.4.2 [45] with the package phyloseq [46]. Unassignable sequences and sequences not belonging to the kingdoms of bacteria or fungi were removed. Unique operational taxonomic unit (OTU) tables were rarefied (set.seed(631)) and low abundances (< 0.1%) removed. DNA samples with number of sequences lower than 50% of the mean number of sequences of all samples per country were removed from the analysis (bacteria: n = 6; fungi: n = 4).

### Soil functions

Microbial soil respiration rates were obtained from a 4.5 g fresh soil sample from each inter-row moisturized to water saturation and measured with a micro-respirometer [47] for about 22 hours and reported as the average consumption of oxygen (μg O_2_/ h * 1 g dry soil) between measurement hours 10–20 h. For estimating decomposition rates, two different organic substrates were used, green tea and rooibos tea from the commercial seller Lipton tea^®^, representing a labile and a recalcitrant substrate, respectively [48]. In adoption of the Teabag Index method [48], the teabags, one of each kind, were buried 8–10 cm deep into the soil at the beginning of the season (April–June), at least three pairs in each vineyard inter-row. The incubation time was about 90 days, except for France where for logistic reasons approximately 35 days were chosen as incubation time. After recollection, the decomposed fraction of the starting weight (%) was calculated for both substrates. Due to the divergence of incubation times between France and the other countries, we decided to use the standardized relative weight loss of both tea types for the comparative analysis rather than to calculate decomposition rates and stabilization factors suggested by the Teabag Index method.

### Soil covariates

From the same soil samples used for DNA extractions, approximately 250 g were used to determine physico-chemical parameters (Table S3 in S1 File). All samples were analyzed at Geisenheim University using standard procedures as described in Schaller (2000) [49]. Soil pH was measured by suspension of soil samples in 0.01 M CaCl₂-solution (1:1.5). The proportion of fine soil organic carbon (OC) and the carbon/nitrogen ratio (C/N) were determined following the Dumas combustion method and using a “Vario MAX CNS” analyzer (Elementar Analysensysteme GmbH, Langenselbold, Germany). To determine the content of OC for calcareous soils (pH > 6.9), the inorganic C fraction was determined, by measuring the calcium-carbonate fraction using the HCl reaction and measuring the volume of released CO_2_. Bioavailable copper (Cu) was determined by extraction using diethylenetriaminepentaacetic acid (DTPA) and was analyzed using an atomic absorption spectrometer. The clay content (< 2 μm soil fraction) was determined as percentage of total fine soil weight by separating clay from the other fractions using a *Köhn* hydrometer, and subsequent drying and weighing. The clay content was determined only for every second sample assuming that neighboring inter-rows have similar clay contents. Determination of soil pH, OC, and C/N ratio was done in 2016 and 2017, clay content and Cu was only determined in 2016, assuming that these parameters are relatively stable across the study years.

### Statistical analyses

#### Microbial alpha diversity and soil functions

Bacterial and fungal richness refer to the sum of unique operational taxonomic units (OTUs) (>97% gene sequence similarity threshold). Fungal and bacterial Shannon-Wiener diversity indices [50, hereafter Shannon Diversity] were calculated for each sample from OTU data. Richness represents the number of observed OTUs, the Shannon diversity weighs species (here OTUs) proportionately to their relative occurrence in the sample (here relative number of sequences), thus, gives information about changes in the dominance structure of species communities.

The different disturbance levels were translated into a numerical gradient for statistical testing: 1 for “low”, 2 for “intermediate, and 3 for “high” disturbance.

All statistical analyses were performed in R version 3.4.2 [45]. The lme4 package version 1.1-18-1 [51] was used for fitting linear mixed effect models (function lmer), using maximum likelihood (ML) to investigate the effect of soil disturbance and soil covariates on microbial diversity (fungal and bacterial richness and Shannon Index) and soil functions (respiration and decomposition). Differences among countries in response to the treatment were tested by including ‘country’ as fixed factor as well as in a 2-way interaction with all other soil covariates. Whether interaction coefficients differed significantly from 0 was determined by applying the sim_slopes function from the package ‘interactions’ [52]. All covariates were standardized by subtracting the mean and dividing by the standard deviation across countries before analyses. The year of sampling and the experimental plot within vineyards were included in the models as nested random factors (1|*vineyard/plot/year*). For soil respiration, the ‘year’ was not included as random factor because only data from one sampling year (2016) was available. Soil respiration was log transformed before analysis to reach normal distribution of residuals. We did not observe relevant deviations from homoscedasticity by visual inspection of model residuals.

The influence of the disturbance gradient on soil covariates was tested (Table S4 in S1 File) to reveal potential confounding factors. Mean values per plot for each soil parameter were used to build linear mixed effect models with disturbance as explanatory variable including ‘year’ nested within ‘country’ as random factors (1|country/year). In some models, the soil parameters Cu, OC and C/N ratio were log transformed to reach a normal distribution of the residuals. The random factor ‘year’ was removed for soil parameters which were analyzed only once during the study. We checked for collinearity between soil covariates by calculating Pearson correlation coefficients and found no correlations above > 0.7, where collinearity of predictor variables distorts model estimations [53]. No variation of any of the soil covariates was significantly explained by the disturbance gradient (Table S4 in S1 File).

#### Microbial community analysis

Microbial community analysis was done using the package vegan version 2.5–4 [54]. Bacterial and fungal data sets were analyzed at the highest taxonomic level that could be resolved with the primers and databases used (mostly at the genus level, although for fungi some OTUs could be assigned at the species level). Analyses were carried out across data sets of all countries (hereafter, the European level) and at the level of each country, considering two consecutive sampling years for Switzerland and Germany but only one for the other countries. All data sets were log-transformed (log10(x+1)) to downweigh very abundant taxa (which were often unresolved taxa of coarse taxonomic levels). Dissimilarity matrices were calculated for all community data sets using Bray-Curtis dissimilarities, which were square-root transformed to avoid negative eigenvalues resulting from eigenvector-based methods [55].

We used permutational multivariate analysis of variance (PERMANOVA) [56] to test the effect of soil disturbance, country, vineyard and year on microbial community composition. The variables ‘disturbance’ and ‘vineyard’ were included simultaneously in the analyses of country-level data sets, except for Switzerland, where these factors were included separately due to the different set-up. ‘Disturbance’ and ‘country’ were included in the analyses of the European level. Further, ‘year’ was tested for Germany, Switzerland, as datasets from two years were available. All analyses were performed with 10 000 permutations.

To visualize microbial community patterns at the European level, principal coordinate analyses (PCoA [57]) were performed on bacterial and fungal community dissimilarity matrices. To constrain the ordination of microbial community patterns by soil covariates, we used distance-based redundancy analysis (db-RDA [55]), including all soil covariates as independent terms and the European level dissimilarity matrices as response term. The db-RDA models were tested using permutation-based ANOVA testing the significance of the model as well as of individual independent terms. Results of PCoA and db-RDA were visualized using the ordination plots showing the first and second axis.

To elucidate the relative importance of disturbance, soil variables, and country of shaping bacterial and fungal communities, variation partitioning [58] based on db-RDA was conducted using three different models containing: (1) soil disturbance, (2) the soil covariates, and (3) country. The adjusted R-squared values can be interpreted as percentages of explained variation of community composition and were reported for each of the three models and their combinations [59]; negative values were considered as zero.

To evaluate which microbial taxa were associated with different disturbance levels, we used the Indicator Value approach on the bacterial and fungal abundance data sets at the European level and for each country [60] Methods, results and discussion of this analysis is given in Appendix 2 in S1 File.

## Results

After preprocessing of the raw sequence data, the final datasets contained in total 8 038 657 bacterial and 14 438 133 fungal sequences representing approximately 47% and 68% of all raw sequences, respectively.

Across all 5 countries, 504 taxa with the finest resolution of genera for bacteria and 916 taxa with the finest resolution of species were obtained including those that were assigned to coarse taxonomic levels or were unassignable. Information about relative abundances of most abundant bacterial phyla and fungal classes can be found in Appendix 3 in S1 File.

Bacterial and fungal richness and Shannon diversity were significantly different among countries (Tables 1 and 2). Average OTU richness per country ranged between 1469 and 2087 for bacteria and between 151 and 444 for fungi (Table 1). The highest richness values for both groups were found in Germany and the lowest in Romania. Average bacterial Shannon diversity per country ranged from 6.22 (AT) to 6.63 (DE) and between 3.16 (FR) and 4.26 (DE) for fungi (Table 1).

**Table 1:** Mean values and standard deviation (sd) of microbial alpha diversity parameters and soil functions for each country (AT, Austria; CH, Switzerland; DE, Germany; FR, France; RO, Romania). Bacterial and fungal richness are represented by the number of OTUs, bacterial and fungal OTU Shannon diversity (shannon), microbial respiration as measured by the consumption of O2 (μg/h), decomposition rates of labile and recalcitrant organic substrate is represented as weight loss of tea bags in %.

Response variables	AT		FR		DE		RO		CH	
	mean	sd	mean	sd	mean	sd	mean	sd	mean	sd
Bacterial OTU richness	1780.94	230.67	1555.77	247.60	2086.55	362.30	1468.87	196.79	1803.62	281.69
Bacterial OTU shannon	6.22	0.19	6.30	0.18	6.63	0.19	6.32	0.14	6.48	0.19
Fungal OTU richness	270.02	56.91	257.08	34.86	443.65	80.59	151.43	58.95	235.76	123.90
Fungal OTU shannon	4.08	0.29	3.16	0.46	4.26	0.32	3.38	0.59	3.55	0.57
Respiration (O_2_ μg/h)	3.41	0.90	2.70	1.04	2.98	0.69	3.41	0.83	4.09	1.66
Decomp. labile substrate (%)	53.05	4.44	59.61	6.42	63.14	5.54	65.41	6.19	57.80	5.32
Decomp. recalcitrant substrate (%)	29.10	5.21	58.56	7.81	35.71	4.81	21.41	9.06	29.31	5.48

**Table 2:** Analysis summary showing Type III Analysis of Variance (ANOVA) summary of general linear mixed models for all included response variables (Resp. var.) and explanatory variables (Expl. Var.).

Resp. var.	Expl. var.	Europe			*Country (Interaction)									
		GLMM coeff	p-Value	sig	AT		FR		DE		RO		CH	
					GLMM Coefficients									
Bacterial OTU richness	disturbance	33.234	0.351		15.19		93.14	.	33.54		13.85		10.45	
	Cu	-40.274	0.045	*	33.32		-58.36		-108.45	*	-33.08		-34.80	
	OC	69.306	0.048	*	-159.50	*	74.18		57.40		272.89	*	101.56	**
	C/N	-46.942	0.189		29.08		-33.90		76.89		-231.22		-75.56	**
	pH	30.564	0.403		-40.84		-62.23	*	149.79		50.62		55.48	
	clay	15.914	0.502		57.92		-76.49		-5.79		154.47	*	-50.54	
	(Intercept)	1637.786	<0.001	***	2052.93	***	1271.99	***	1973.11	***	1182.50	***	1708.41	***
Bacterial OTU shannon	disturbance	0.025	0.169		0.02		0.07	**	0.01		0.01		0.01	
	Cu	-0.035	0.008	**	0.05	.	-0.02		-0.07	*	-0.03		-0.10	***
	OC	0.057	0.017	*	-0.10	*	0.06		0.01		0.23	*	0.08	***
	C/N	-0.047	0.047	*	0.01		0.00		0.03		-0.22	*	-0.05	**
	pH	0.017	0.493		-0.08		-0.06	**	0.12		0.09	**	0.02	
	clay	-0.006	0.687		0.03		-0.10	*	-0.01		0.09	.	-0.04	
	(Intercept)	6.347	<0.001	***	6.36	***	6.15	***	6.54	***	6.21	***	6.46	***
Fungal OTU richness	disturbance	2.270	0.845		-32.95	*	16.76		15.86		31.29	*	-19.61	
	Cu	2.728	0.638		28.15	**	-3.71		8.82		-21.86		2.24	
	OC	3.322	0.737		-37.98	*	18.73		10.01		-1.95		27.80	**
	C/N	1.202	0.905		9.91		2.26		23.96		-6.01		-24.11	**
	pH	-17.448	0.097	.	-83.51	**	-22.88	**	18.88		-19.24		19.51	
	clay	16.444	0.016	*	44.60	***	7.68		2.98		17.25		9.71	
	(Intercept)	264.984	<0.001	***	319.74	***	233.62	***	444.82	***	99.36	***	227.37	***
Fungal OTU shannon	disturbance	-0.048	0.264		0.01		-0.09		-0.05		0.03		-0.14	*
	Cu	-0.015	0.680		0.10		0.01		0.03		-0.18		-0.02	
	OC	-0.016	0.806		0.00		-0.15		-0.10		0.10		0.07	
	C/N	0.102	0.113		0.08		0.16	.	0.04		0.26		-0.03	
	pH	-0.139	0.028	**	-0.45	*	-0.18	**	0.12		-0.17	*	-0.01	
	clay	0.152	<0.001	***	0.05		0.27	*	0.04		0.33	*	0.08	
	(Intercept)	3.580	<0.001	***	4.20	***	2.89	***	4.19	***	3.03	***	3.59	***
Microbial respiration	disturbance	-0.069	<0.001	***	-0.11	***	-0.09	***	0.07	*	-0.06	*	-0.17	***
	Cu	-0.010	0.532		-0.02		-0.12	***	0.02		-0.04		0.10	**
	OC	0.259	<0.001	***	0.26	***	0.27	***	0.45	***	0.07		0.25	***
	C/N	-0.092	<0.001	***	-0.14	***	-0.07	***	-0.18	**	-0.01		-0.06	
	pH	-0.053	0.118		-0.09		-0.05	*	-0.15		-0.04		0.05	
	clay	0.016	0.331		-0.03		-0.11	*	0.02		0.11	*	0.08	*
	(Intercept)	1.142	0.001	**	1.24	***	1.02	***	1.23	***	1.14	***	1.07	***
Decomposition labile substrate	disturbance	0.346	0.311		0.72		2.53	***	-2.08	**	0.60		-0.03	
	Cu	0.850	0.061	.	-0.39		-1.83	*	1.21		1.47		3.79	***
	OC	-1.127	0.136		0.72		-0.21		-1.77		-5.23	.	0.86	
	C/N	1.479	0.093	.	-1.91	.	1.70	*	1.08		8.67	*	-2.14	*
	pH	1.147	0.226		0.94		1.01		5.62		-1.90	*	0.06	
	clay	1.039	0.027	*	-0.35		-1.53		1.02		5.21	***	0.84	
	(Intercept)	57.641	0.032	*	54.23	***	61.21	***	59.36	***	59.38	***	54.03	***
Decomposition recalcitrant substrate	disturbance	1.330	0.002	**	2.40	*	0.69		-0.71		1.87	*	2.39	**
	Cu	-0.015	0.977		0.39		-1.15		1.22		1.40		-1.94	.
	OC	0.788	0.399		0.72		0.64		-1.27		1.98		1.88	*
	C/N	1.914	0.058	.	0.07		-0.11		2.74		3.22		3.65	**
	pH	-0.157	0.886		-0.10		0.10		0.88		-2.39	*	0.73	
	clay	1.373	0.013	*	-2.12	*	-0.79		1.18		9.06	***	-0.46	
	(Intercept)	33.810	<0.001	***	29.75	***	58.84	***	34.52	***	16.15	***	29.81	***

GLMM coefficients were calculated from Tables S5–S8 & S11–S13 in S1 File. We investigated the effect of plot disturbance and other soil covariates on fungal and bacterial OUT richness and Shannon diversity (Shannon), microbial respiration and decomposition of labile and recalcitrant substrate. Plot nested within vineyard and year was included in the models as random effect (1|vineyard/plot/year) and country (AT, Austria; CH, Switzerland; DE, Germany; FR, France; RO, Romania) was included as an interaction term for all analyses (‘***’ significant at p < 0.001; ‘**’ significant at p < 0.01; ‘*’ significant at p < 0.05; ‘.’ Significant at p < 0.1). Significances of the interaction coefficients from 0 were obtained by using the R package”interactions”[52].

### Effects of soil disturbance and covariates on microbial alpha-diversity

Soil disturbance did not significantly affect bacterial richness, neither across nor within countries (Table 2, Table S5 in S1 File). Bacterial Shannon diversity was not affected by soil disturbance across countries (Table 2, Table S6 in S1 File), however, for vineyards in France, a significant positive effect of soil disturbance on bacterial diversity was evident.

Soil copper content had a significant negative effect on bacterial richness and Shannon diversity at the European level (Table 2). In four out of five countries, soil copper content also had a negative effect on bacterial richness and diversity, which was, however, not significant in all cases. An exception was Austria, with copper showing no negative effects on soil bacterial richness and diversity. Soil organic carbon significantly increased bacterial richness and Shannon diversity in vineyard soils at the European level, which was supported by positive slopes within most countries except for Austria, where the influence was negative on bacteria. Soil C/N ratios negatively affected bacterial Shannon diversity at the European level. Soil C/N ratios, pH and clay content had inconsistent effects on soil bacterial diversity within different countries (Table 2, Table S7 in S1 File).

Neither fungal richness nor fungal Shannon diversity were affected by soil disturbance at the European level. However, within countries fungal richness increased with increasing disturbance in Romania but decreased in Austria (Table 2, Table S7 in S1 File). There was no significant effect of soil disturbance on fungal richness in vineyards in the other countries (Table 2, Table S8 in S1 File).

Increasing soil pH affected fungal Shannon diversity negatively at the European level while an increasing soil clay content was positively associated with fungal richness (16.44, p = 0.016) and fungal Shannon diversity (0.152, p <0.001). Effects of soil variables on soil fungal richness and diversity within countries were not consistent and indicated positive and negative effects (Table 2, Tables S7 & S8 in S1 File).

### Effects of soil disturbance on microbial community composition

At the European level, soil disturbance explained less than 1% of bacterial and fungal community variation between sites (bacteria R^2^ = 0.007, fungi R^2^ = 0.007, Table 3). At the level of countries, the proportions of variation explained by soil disturbance were slightly higher compared to the European level: Values for soil bacteria and fungi ranged from about 3% (Switzerland, bacteria R^2^ = 0.028, fungi R^2^ = 0.026) to about 6% (France, bacteria R^2^ = 0.058, fungi R^2^ = 0.064). PERMANOVA permutation tests for soil disturbance were significant for all cases, except for the country-level analysis of Romania (bacteria R^2^ = 0.037, p = 0.091; fungi R^2^ = 0.036, p = 0.26).

**Table 3:** Results of PERMANOVA of bacterial and fungal communities at European and country level (AT, Austria; CH, Switzerland; DE, Germany; FR, France; RO, Romania).

Variable	Group	Data set	R^2^	P-value	
Country	Bacteria	Europe	0.329	0.0001	***
	Fungi	Europe	0.179	0.0001	***
Disturbance	Bacteria	EUROPE	0.007	0.0006	***
		AT	0.048	0.0004	***
		CH^1^	0.028	0.0001	***
		DE	0.040	0.0001	***
		FR	0.058	0.0001	***
		RO	0.037	0.0910	
	Fungi	Europe	0.007	0.0002	***
		AT	0.040	0.0073	**
		CH^1^	0.026	0.0001	***
		DE	0.029	0.0001	***
		FR	0.064	0.0001	***
		RO	0.036	0.2597	
Vineyard	Bacteria	AT	0.290	0.0001	***
		CH^1^	0.283	0.0001	***
		DE	0.143	0.0001	***
		FR	0.265	0.0001	***
		RO	0.244	0.0001	***
	Fungi	AT	0.313	0.0001	***
		CH^1^	0.345	0.0001	***
		DE	0.179	0.0001	***
		FR	0.376	0.0001	***
		RO	0.284	0.0001	***
Year	Bacteria	CH	0.072	0.0001	***
		DE	0.145	0.0001	***
	Fungi	CH	0.068	0.0001	***
		DE	0.035	0.0001	***

For the European data sets, effects of soil disturbance and country were analyzed. For the country level data sets, the effects of disturbance, year, and vineyard were analyzed where applicable. R2: partial R2; P-value based on 9999 permutations (‘***’ significant at p < 0.001; ‘**’ p < 0.01; ‘*’ p < 0.05; ‘.’ p < 0.1).

^1^ Due to the experimental design of CH, vineyard and disturbance needs to be separated for PERMANOVA

Differences among countries explained 33% and 18% of variation of the European soil bacterial and fungal community data, respectively (bacteria R^2^ = 0.329, fungi R^2^ = 0.179; Table 3). The effect of vineyard explained between 14% and 29% of variation of the country-level bacterial community data, and between 18% and 38% of variation of the country-level fungal community data. The effects of country and vineyards were significant in all cases.

For countries with two sampling years, the effects of year on microbial community dissimilarities were significant. For bacteria, year explained 7% of variation in Switzerland (R^2^ = 0.0719) and 15% in Germany (R^2^ = 0.145). For fungi, year explained 7% of variation in Switzerland (R^2^ = 0.068) and 4% in Germany (R^2^ = 0.035).

### Patterns of microbial community composition

**Fig 1:**
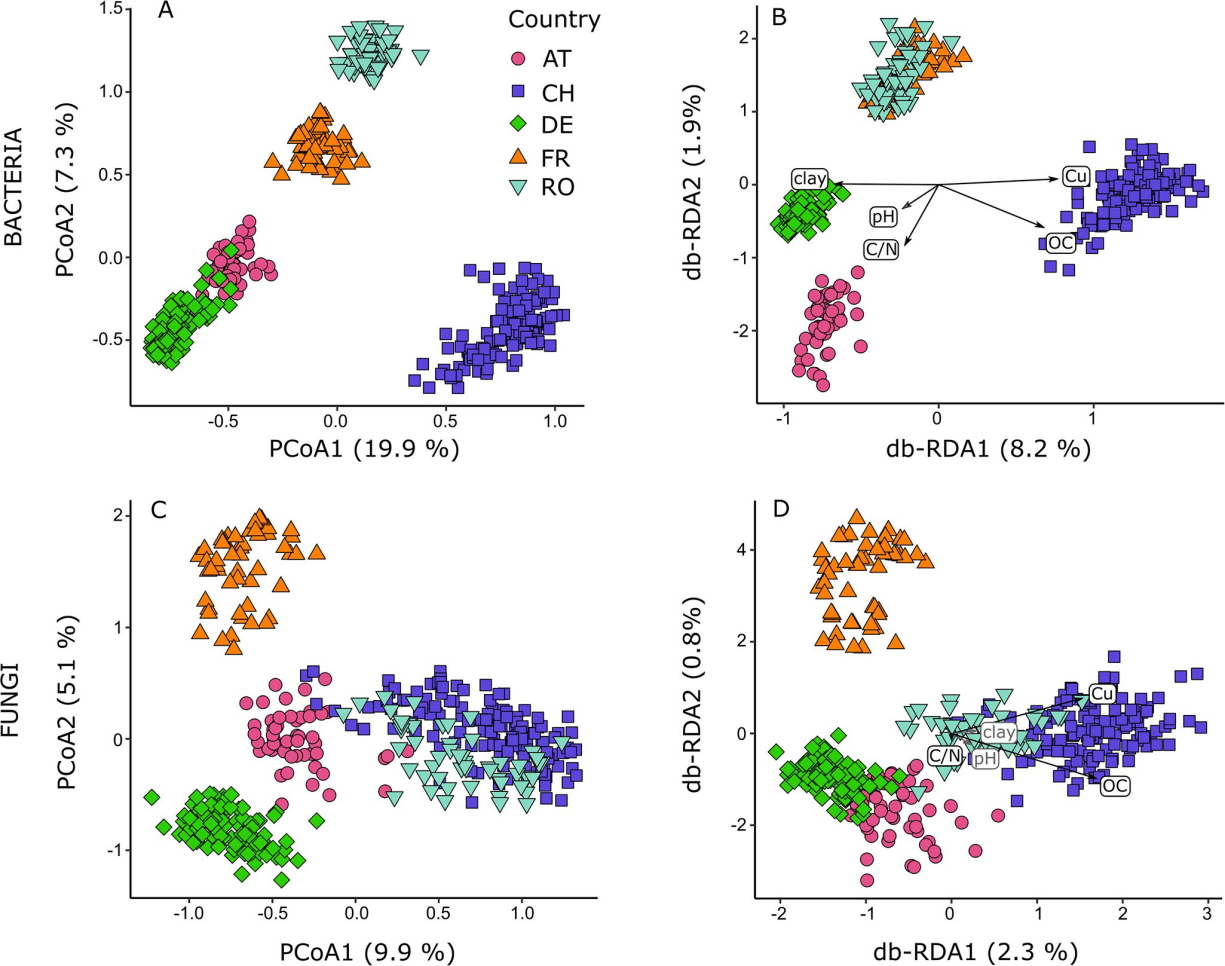
Results of community analysis of vineyard soil bacteria (A, B) and fungi (C, D): Left panels (A, C) showing principal coordinate analysis (PCoA) and right panels (B, D) distance-based redundancy analysis (db-RDA). Directions of black arrows indicate gradient of constraining soil variables (OC: organic carbon; C/N: carbon/nitrogen ratio; Cu: bioavailable soil copper content; pH: soil pH; clay: clay content). Symbol colors and shape indicate countries (pink circle: AT, Austria; purple squares: CH, Switzerland; green diamond: DE, Germany; orange triangle: FR, France; light blue inverse triangle: RO, Romania). Axis labels indicate percentage of explained variance by axis.

Applying PCoA, soil bacterial communities formed five separated clusters that largely corresponded to countries (Fig 1A). All clusters were well separated except for the Austrian and German cluster, which showed some overlap. Along the first ordination axis, the German and Swiss clusters were most distant with Austrian, French, and Romanian samples located in between. Along the second axis, the Romanian cluster was most distant from the German and Swiss clusters.

For soil fungal communities, the Austrian, German, and French samples assembled at the country-level (Fig 1C), while the Swiss and Romanian samples formed a combined cluster. Along the first ordination axis, the German and the Swiss/Romanian clusters were located most distant with the Austrian cluster being located in between and showing small overlap with the Swiss/Romanian cluster. Along the second axis, the French cluster was located most distant from the German cluster, while the other clusters were located in between.

Distance-based RDA was performed for soil bacteria and fungi using the soil covariates as constraining variables (Fig 1B and 1D, Table S13 in S1 File). For bacteria, the first axis was dominated by the gradients of soil Cu, OC, and clay contents, the second axis by the gradients of soil pH and C/N ratio. Austrian and German samples were associated with high clay, but lower soil OC and Cu content. Swiss samples were associated with high soil OC and Cu, but low clay content. In contrast to the PCoA results, Romanian and French samples formed one cluster, which was associated with low soil C/N ratio and low soil pH values.

For fungi, the effect of soil pH on fungal community composition was not significant (Fig 1D, Table S13 in S1 File). Further, indicated by short arrow lengths, the effects of clay content and C/N ratio were neglectable. The first axis was dominated by the gradients of soil Cu and OC content, which drove the separation of the Swiss/Romanian cluster (high Cu and OC) from the Austrian/German cluster (low Cu and OC). French samples were separated from the other clusters by the second axis.

**Fig 2:**
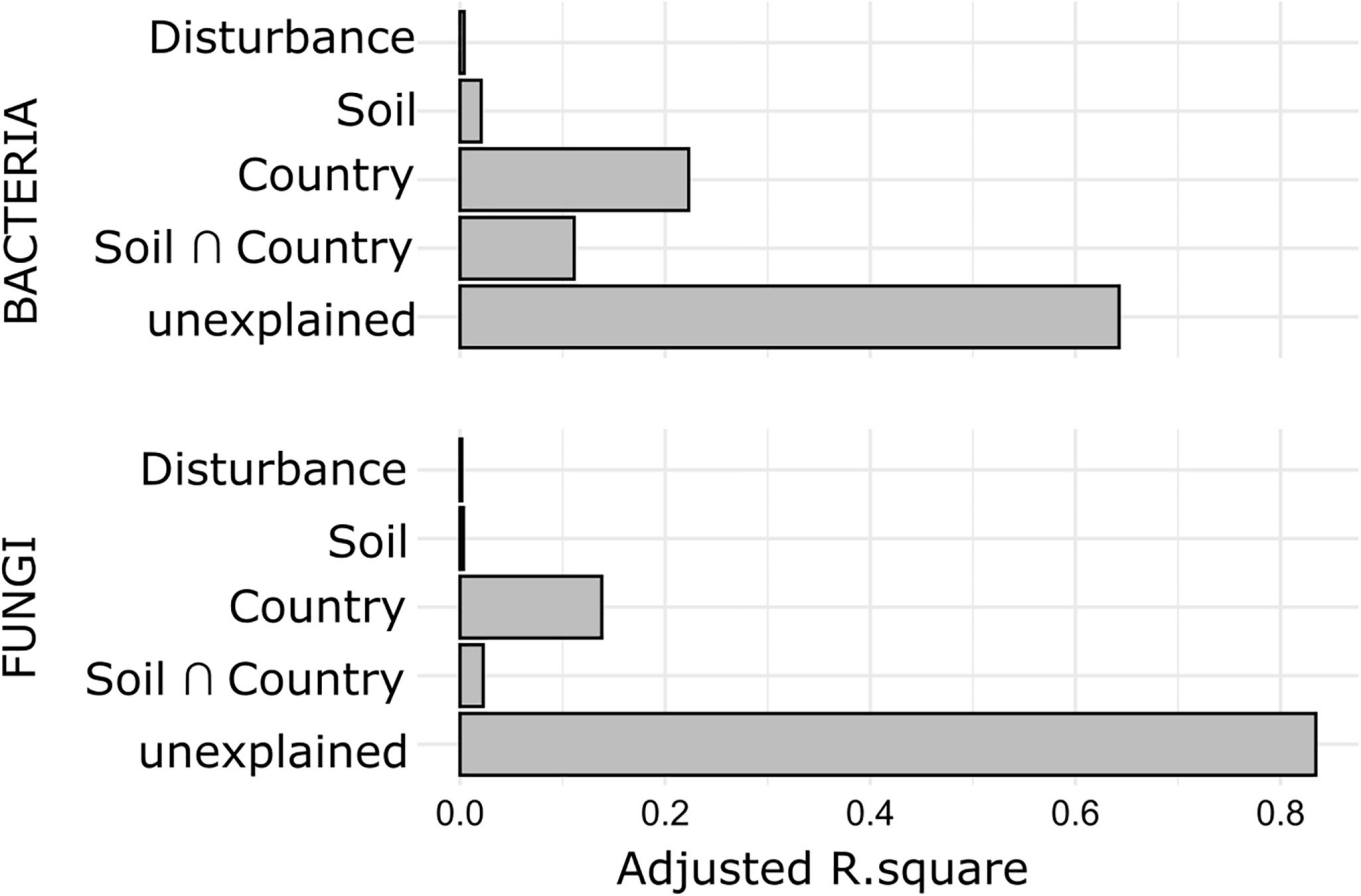
Variance partitioning based on db-RDA of vineyard soil bacterial and fungal communities. Adjusted R-squared values expressing relative amount of explained variation of communities by disturbance, soil covariates, country, as well as the intersection of soil variables and country. Fractions with adjusted R-squared values of zero not shown.

Variation partitioning of the European bacterial community revealed that 22% of community variation was explained solely by country, 11% of variation was explained by the intersection of fractions of country and soil variables, a fraction of 2% of variation was explained uniquely by soil variables. Less than 1% was explained by the unique effect of soil disturbance (Fig 2A). About 64% of bacterial community variation was unexplained.

For the European fungal community data, 14% of community variation was explained solely by country (Fig 2B, Table S14 in S1 File) and 2% of variation was explained by the intersect of fractions of country and soil covariates. The unique effect of soil covariates and soil disturbance explained less than 1% of total fungal community variation. About 83% of variation remained unexplained.

### Effects of soil disturbance on soil functions

In European vineyards, average soil microbial respiration rates ranged between 2.70 μg O_2_ /h (FR) and 4.09 μg O_2_ /h (CH) (Table 1). Soil disturbance consistently reduced respiration of microorganisms at the European level (Table 2). The significant negative effect of soil disturbance on microbial respiration was also detected within most countries except in Germany, where respiration was increased with increasing soil disturbance (Table 2, Table S10 in S1 File). Organic carbon affected microbial respiration positively and high C/N ratios had a negative effect across and within countries, although this effect was not consistently significant within countries (Table 2, Table S10 in S1 File). Soil copper, pH and clay content had no effect at the European level and showed inconsistent but significant positive and negative effects within some countries (Table 2, Table S10 in S1 File).

Average decomposition rates of labile substrate ranged between 53.05% (AT) and 65.41% (RO) (Table 1). The average decomposition rate of recalcitrant substrate was lower and ranged between 21.41% (RO) up to 35.71% (DE). In French vineyards decomposition rate of recalcitrant substrate was highest with 58.59% (Table 1). The two substrate types were differently affected by soil disturbance. Decomposition rates of labile substrate were not affected by soil disturbance across countries. However, within countries, soil disturbance had a negative effect on decomposition rates of labile substrate in German (Table 2, Table S11 in S1 File) but a positive effect in French vineyards (Table 2; for the other countries no pattern was detected). Soil disturbance increased decomposition rates of recalcitrant substrate at the European level and this trend was significant for data obtained in three countries (Table 2, Table S12 in S1 File). High proportions of clay in vineyard soils significantly increased the decomposition of both substrates across countries (Table 2, Tables S11 & S12 in S1 File).

## Discussion

### Effects of soil disturbance on bacterial and fungal diversity

By setting-up a European study design spanning five countries combined with within-country replication realized by nine study sites, we aimed to examine if responses of microbial diversity to soil disturbance could be generalized across larger environmental and spatial gradients [61]. We assumed that soil disturbance represented by tillage or herbicide application represent such a strong impact on soil microbial communities that patterns of changes of diversity of soil bacteria and fungi should emerge at the European scale, overriding the effects of local variations of soil conditions and the biogeographic patterns to at least some extent. However, for our data, this hypothesis must be rejected. Soil disturbance did neither consistently increase nor decrease alpha-diversity of bacterial and fungi at the European level. Also, effects of soil disturbance on soil bacterial and fungal community composition were minor compared to soil variables and other site specific effects.

At the level of countries, a few significant effects of soil disturbance on bacterial and fungal OTU richness and Shannon diversity were evident, yet they were not consistent between countries and, sometimes, had opposite directions. For example, soil disturbance by tillage had a negative effect on fungal richness in Austria, but a positive effect in Romania. Results from previous studies, usually restricted to one or few experimental fields, were similarly inconclusive reporting, on one hand, an increase of bacterial richness in response to tillage [20, 36], or, on the other hand, a decrease of bacterial richness [17, 62]. For fungal richness, positive effects of no-tillage or conservation tillage treatments were found in other studies [36, 63].

Soil parameters are among the most important drivers of alpha-diversity of soil bacteria [8] and fungi [9]. For our data, we found several effects of soil parameters at the country-level and at the European level, e.g., a negative effect of Cu on bacterial richness and a positive effect of clay on fungal richness. It is possible that the effects of soil parameters on soil microbial communities have masked the effects of soil disturbance [64].

Regarding the composition of microbial communities, soil disturbance introduced by soil management practices explained less than 1% of bacterial and fungal community composition at the European level, but up to 6% at the level of countries. This number is consistent with other studies that investigated the effects of tillage on soil microbial communities [5, 11, 65, 66], suggesting that our country-level results were in the expected range of variation of microbial community shifts. In addition, our results confirmed that effects of soil disturbance depend on the spatial scale (local vs. continental [7]). Similar to our study, Burns et al. [11] found that the effects of tillage on soil microbial community composition within the regional subsets of vineyards were higher compared to the full set of sites, concluding that the effects of tillage depend on local edaphic conditions. It also corroborates to the finding that for soil bacteria cropping system has the weakest effect compared to soil properties and geographic location if large geographic ranges are investigated [67].

Analysis of community patterns showed a relatively large influence of the vineyard level with pseudo-R^2^-values 0.14 and 0.37. This indicates that bacterial and fungal community composition varied considerably between vineyards. These differences in community composition might have led to inconsistent responses to disturbance because taxa are differently affected by disturbance events. Lacking a consistent effect of disturbance by tillage or herbicide application even at the country level, we conclude that metrics of soil microbial diversity are less consistently affected than it may appear from single-location studies.

Our results support the widely accepted view that soil microbial communities are shaped by soil parameters [8, 68, 69]. However, regarding the most important soil variables determining bacterial and fungal community composition, our results only partly corroborate previous studies. For soil bacteria, we found that copper, clay and SOM were the most important soil variables at the European level, while the soil pH, played a less important role. Soil pH was regularly found to be the most important driver of bacterial community patterns in other large scale studies [8, 69], which both showed a high correlation of bacterial alpha diversity as well as community composition with soil pH covering a gradient from 4 to 9. However, our sites covered only a gradient of soil pH ranging from 5.1 to 7.6 depending on country. In contrast to soil pH we covered long gradients of clay content (0–37%) and bioavailable Cu (1.5–520 mg/kg). These gradients were sufficient to discover strong effects on bacterial and fungal communities.

We identified several taxa that were associated with certain disturbance levels at the country level using the Indicator Value approach. However, we only found 4 bacterial and 3 fungal taxa that were identified as indicators for different disturbance levels in more than one country. This is a low number, given that we found a total number of 504 bacterial and 916 fungal taxa across all countries. This corroborates our conclusion that responses of microbial communities in one region might not be valid for other regions because of different local community composition and environmental conditions. For a comprehensive discussion of the Indicator Value analysis, see Appendix 3 in S1 File.

### Factors shaping microbial community composition at the European level

A high proportion of microbial community variation was explained by soil covariates and the country, namely about 30% of variation of soil bacterial communities and about 18% of variation of fungal communities. Accounting for soil disturbance and soil covariates, a large part of variance of microbial community composition of bacteria and fungi was attributed to the country level. This indicates a high distinctiveness of microbial community composition at the country level. This might be an indication for the action of biogeographic processes (e.g. historical dispersal and extinction dynamics, diversification or drift events [6, 70]) leading to distinct microbial communities at different locations (in contrast to the “everything-is-everywhere” hypothesis [71]).

Additionally, various country-specific covariates may have acted as confounding factors. Vineyards are intensively managed agro-ecosystems viticultural management is accompanied by many factors, which are sometimes highly specific at the regional level (e.g., the use of certain pesticides, cultivars, irrigation). Moreover, the age and history of vineyards as well as previous soil management practices, could be site-specific and are often difficult to reconstruct retrospectively. Our experimental design is not suited to partition all these effects, as our study focus was to investigate the effects of soil disturbance by tillage org herbicide application across a regional and European scales.

### Effects of soil disturbance on microbial functions

The influence of disturbance on soil functions was heterogeneous. Microbial respiration was negatively affected by disturbance in vineyards in four out of five countries. This could be due to either a lower microbial abundance or a lower microbial activity with increasing disturbance. Disruption of inter-relationships between microbes and root exudates from inter-row vegetation cover [72, 73] or indirect effects of tillage on soil organic carbon dynamics are known to affect carbon use efficiency of microorganisms [13]. The consistency of the respiration patterns highlights the positive influence of above-ground vegetation on soil microbial respiration [74, 75]. Microbial respiration is generally used as a soil quality indicator. It is linked to nutrient mineralization processes and to nutrient turnover [76] and a change in microbial respiration can result in altered nutrient provision for crops [33, 74, 77].

While decomposition of labile substrate did not show a general pattern, the decomposition rates of recalcitrant substrate were higher in disturbed than in undisturbed soils across European countries. Different substrates are decomposed by different microbial taxa [78, 79]. The decomposition performance of communities can be influenced by the past local resource availability [80, 81]. There is a relatively high deposition of recalcitrant material such as woody pruning material, leaves or pomace in vineyards. The removal of the vegetation cover in vineyard plots by tillage or herbicide application likely caused a reduced availability of labile substrate like root exudates. Thus, the predominant resource for decomposers were mainly recalcitrant substrates like woody plant material. The increase in decomposition of recalcitrant substrate in disturbed vineyards in our study could therefore be due to microbial communities that might have become more specialized [77, 78] on the decomposition of recalcitrant material.

In most cases, respiration rates were higher in less disturbed soils. It is likely, that the vegetation cover in these soils provided more labile substrate, which may have mediated a more active microbial community compared to highly disturbed soils. This may alter mineralization rates of nutrients for crop plants [82, 83].

## Conclusion

We found little and inconsistent effects of vineyard soil disturbance on soil microbial alpha-diversity and soil functions across five European countries. As well, soil microbial community composition was little affected by soil disturbance at the European scale. In some countries, the effect of soil disturbance was more pronounced compared to the European level, and sometimes pointed in different directions. Therefore, we conclude that it is difficult to extrapolate results from one locality to others because microbial communities and environmental conditions vary strongly over larger geographical scales. Considering our results, we advocate for small-scale studies using replications at locally separated fields to account for spatial variation of independent and response variables. For future studies aiming at identifying soil management practices fostering soil microbial diversity, we recommend to focus on local wine-growing regions.

## Supporting information

S1 File (PDF)
