# Supplementary material for: Local conditions matter: Minimal and variable effects of soil disturbance on microbial communities and functions in European vineyards
Source: PLoS One. 2023 Jan 27;18(1):e0280516. doi: 10.1371/journal.pone.0280516 (PMC9882891; doi:10.1371/journal.pone.0280516)
Supplement: S1 File — (PDF) [file pone.0280516.s001.pdf]

Supporting information

Appendix 1 Soil sampling, DNA extraction, sequencing, bioinformatics and sequence preprocessing

In each country, soil samples were taken at least one season after experimental plot preparation (2016 and/or 2017) and at minimum 6 weeks after tillage/herbicide application around the time of grapevine flowering. Eight subsamples were taken in the two central inter-rows of each plot to a depth of 10 cm approximately 30 – 50 cm apart from each other and were pooled afterwards into one mixed sample, summing up to a total of 405 soil samples. The number comprises n= 54 samples for Austria 2016, France 2017, Romania 2017, Germany 2016 and Germany 2017, but n = 77 for Switzerland 2016 and n = 58 for Switzerland 2017. Thus, we included samples for bacteria and fungi for each country for at least one season (2016 for Austria, 2017 for France and Romania) or two seasons for Switzerland and Germany. DNA extraction from each soil sample was either performed directly after soil sampling or samples were stored frozen at -20° C until extraction. DNA was extracted from 0.25 g of mixed soil sample per inter-row by using the DNeasyPowerSoil Kit® following the manufacturer's protocol (QIAGEN N.V., Venlo, Netherlands).

DNA sequencing was conducted for the bacterial V4 region of the 16S rDNA using the primers 515f: 5'-GTGYCAGCMGCCGCGGTAA-3' and 806rB: 5'-GGACTACNVGGGTWTCTAAT-3' (1), and for the fungal Internal transcribed spacer ITS2 using the primer pair ITS4: 5'-TCCTCCGCTTATTGATATGC-3' and fITS7: 5'-GTGARTCATCGAATCTTTG-3' (2, 3). Sequencing of 250bp paired-end amplicons was conducted on a MiSeq Illumina machine at Génome Quebec Innovation Centre (Montreal, Canada).

Raw Illumina fastq reads were quality controlled with FastQC (4), generally showing good quality. The reads were cleaned and filtered using sickle (5).

For Bacteria, the software package Mothur (version 1.39.5) was used for sequence analysis (6) while following the Standard Operating Procedure outlined on http://www.mothur.org/wiki/MiSeq_SOP.

Briefly, the two overlapping paired-end reads were combined using make.contig. Then, each unique sequence was aligned with align.seqs to the SILVA reference alignment release 132 (7). A distance matrix was calculated allowing for four mismatches. Chimeric sequences were identified using chimera.uchime and removed. Sequences matching "Chloroplast-Mitochondria-unknown-Archaea-Eukaryota" were also removed. Next, sequences were clustered using the opticlust clustering algorithm (8) to build operational taxonomic units (OTUs). The resulting file was parsed to separate the data for each sample. OTUs were assigned a taxonomic group with classify.seqs using the RDP reference file release 11 (9) and a cut-off of 80% of the bootstrap value. For the description of the community, the sequences are split at the order level and OTUs with the same taxonomy were clustered together at cut-off level 0.03. Mothur was used to convert data into biom format files.

Sequence analysis for fungi was conducted using the software package PIPITS (version 1.3.x, (10)). This was necessary because Mothur relies on alignment of all sequences which is not possible for fungi. PIPITS generates a biom file for OTU with the UNITE fungal ITS reference set based on the RDP classifier (11).

Downstream sequence preprocessing was done in R version 3.4.2 (12) with the package phyloseq (13). Unassignable sequences not belonging to the kingdom of bacteria or fungi were removed. OTU tables were rarefied (set.seed(631)) and low abundances (< 0.1%) removed. DNA samples with number of sequences lower than 50 % of the mean number of sequences of all samples per country were removed from the analysis (bacteria: n = 6; fungi: n = 4).

Appendix 2: Microbial taxa responding to different disturbance levels

Methods

To evaluate which microbial taxa were associated with different disturbance levels, we used the Indicator Value approach on the bacterial and fungal abundance data sets at the European level and for each country (14). The approach allows to identify taxa with a preference or sensitivity to a certain disturbance level. The Indicator Value shows associations of taxa with samples belonging to certain

target groups (in our case different disturbance levels) by calculation of specificity, which signifies the probability of the site belonging to the target group, given that the taxon has been at that site, and sensitivity, which signifies the probability of a taxon occurring at a site of the target group. We conduct the Indicator Value analysis using the multipatt function of the indicspecies package (14). We tested all soil disturbance levels as target groups including their combinations and applied permutation tests (n = 10000) using 'vineyard' as block variable to test for significance of associations between taxa and groups. We only reported taxa with Indicator Values > 0.6 and p < 0.05.

Results

Using the Indicator Value approach, we identified two bacterial genera but no fungi that were associated with soil disturbance at the European level (Table S9). The bacterial genera *Hymenobacter* and *Pontibacter* were identified as indicator taxa for the combined disturbance levels 'intermediate + high', suggesting that these genera were less abundant in soils of low disturbance.

At the level of countries, we found various taxa associated with different disturbance levels (Table S9). Here, we only report taxa that show a consistent pattern in more than one country . Four bacterial taxa and three fungal taxa were assigned as indicators for disturbance levels in more than one country, however, no taxon was found that was assigned as indicator in all countries. The bacterial genus *Dyadobacter* was associated with the combined disturbance level 'low + intermediate' in Austria and Switzerland, indicating that *Dyadobacter* were sensitive too high disturbance. The bacterial genera *Hymenobacter*, *Pontibacter* (in accordance with the European level) and the assemblage of unclassified members of the phylum *Armatimonadetes* were identified as indicators for the combined disturbance level 'intermediate + high' in Austria, Germany, and France.

The fungal family *Glomeraceae* was identified as indicator taxon for the combined disturbance level 'low + intermediate' in Austria and Germany suggestion susceptibility of this family to high disturbance.

The species *Sarocladium strictum* was associated with disturbance level ‘low + intermediate’ in Germany and France. The species *Spizellomyces dolichospermus* was an indicator for the combined soil disturbance ‘intermediate + high’ for Germany and France.

Discussion

The Indicator Value approach evaluates the predictive power of taxa for different habitats (in our case soil disturbance levels). The approach is suitable to find taxa that show contrasting patterns between different habitats rather than identifying small changes of taxa abundances (14). At the level of individual countries, we identified several taxa with a prediction power for different disturbance levels. This shows that the changes in community composition indicated by PERMANOVA was accompanied by strong changes of abundances of individual taxa.

However, we only found 4 bacterial and 3 fungal taxa that were identified as indicators for different disturbance levels in more than one country. This is a low number, given that we found a total number of 504 bacterial and 916 fungal taxa across all countries. This corroborates our conclusion that responses of microbial communities in one region cannot be simply extrapolated to other regions because of different local community composition and environmental conditions.

The bacterial genus *Dyadobacter* was identified as indicator for low and intermediate disturbance, i.e., it is rare in highly disturbed soils. The genera *Pontibacter* and *Hymenobacter* as well as the group of unclassified *Armatimonadetes* were associated with samples under intermediate to high soil disturbance. The functions of these bacteria, commonly found in soil, are largely unknown. However, the phylum *Armatimonadetes* has previously been found to be positively correlated with tilled soils and negatively with cover crops in Californian vineyards (15).

The fungal family *Glomeraceae*, whose members are known to interact with plant roots forming arbuscular mycorrhiza, was associated with low to intermediate disturbed soils. It is known that arbuscular mycorrhizal fungi are susceptible to tillage because of disruption of mycelial networks (16).

Similarly, the application of Glyphosate caused a decline of arbuscular mycorrhizal fungi in green house experiments (17). Grapevines also benefit from mycorrhizal symbiosis (16), thus, supporting Glomeromycotan diversity by reducing soil disturbance practices might be beneficial for grapevine growth.

The fungal species *Sarocladium strictum* and *Spizellomyces dolichospermus* were also identified as indicator species. While *S. dolichospermus* indicated intermediate to highly disturbed soils, *S. strictum*, which is known as a plant pathogen in many crops (18), was associated with low to intermediate disturbed soils.

Appendix 3 Relative abundances of taxa

The most abundant bacterial phyla (> 1% total relative abundance) were *Acidobacteria* (~30%), *Proteobacteria* (~21%), *Actinobacteria* (~14%), *Planctomycetes* (~5%), *Bacteroidetes* (~4%), *Verrucomicrobia* (~3.5%), *Chloroflexi* (~1.5%), *Gemmatimonadetes* (~1.3%) and unclassified *bacteria* (~17%).

The most abundant fungal orders (> 1% total relative abundance) were *Hypocreales* (~18%), *Pleosporales* (~11%), *Sordariomycetes* (~9%), *Helotiales*(~5.1%), *Dothideomycetes* (~4%), *Xylariales* (~3.3%), *Mortierellales* (~4.4%), *Tremellales* (~3.2%), *Sordariales* (~3.2%), *Agaricales* (~2%), *Capnodiales* (~1.8), *Chaetothyriales* (~1.5%), *Pezizales* (~1.3%). Sequences not assignable to order level, like *Ascomycota* (~11%) and 'unclassified fungi' (~13%) did also contribute to a relevant extent to fungal community composition.

Appendix 4 Supplementary figure and tables

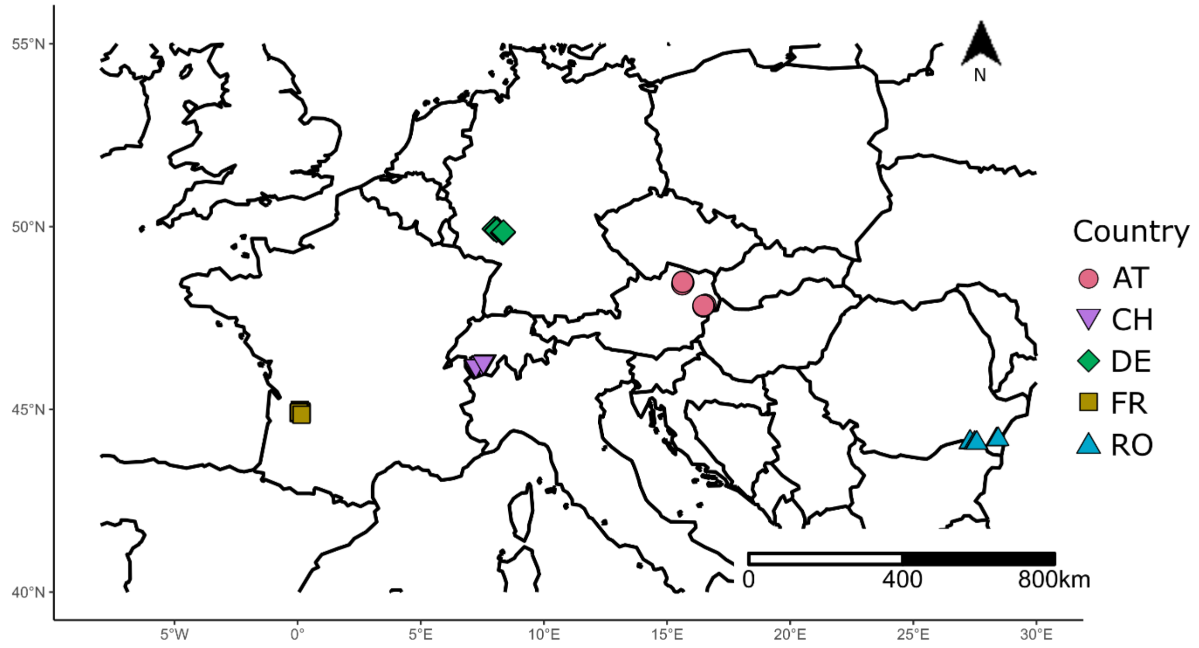

Fig. S1 Map showing localities of vineyards in vine-growing regions of five different countries (red circle: AT, Austria (Kamptal, Kremstal, Leithaberg); purple inverse triangle: CH, Switzerland (Valais); green diamond: DE, Germany (Rheinhessen); ocher square: FR, France Bordeaux, Libournais; blue triangle: RO, Romania (Dobrogea))

Supplementary tables

Table S1 Details of vineyards used for the study.

Table S2 Implementation of vineyard soil disturbance levels "low", "intermediate", "high" in the countries involved in the study.

Table S3 Ranges, mean values and standard deviations of soil covariates.

Table S4 Summary of general linear mixed model fit by maximum likelihood for each soil covariate.

Table S5 Summary of coefficients from linear mixed effect model fitted by maximum likelihood for bacterial OTU richness.

Table S6 Summary of coefficients from linear mixed effect model fitted by maximum likelihood for bacterial OTU Shannon Index.

Table S7 Summary of coefficients from linear mixed effect model fitted by maximum likelihood for fungal richness fungal OTU richness.

Table S8 Summary of coefficients from linear mixed effect model fitted by maximum likelihood for fungal fungal OTU Shannon Index.

Table S9 Results of Indicator species analysis of bacterial and fungal communities at European and country level.

Table S10 Coefficients from linear mixed effect model fitted by maximum likelihood for microbial respiration.

Table S11 Summary coefficients from linear mixed effect model fitted by maximum likelihood for decomposition of labile substrate.

Table S12 Summary coefficients from linear mixed effect model fitted by maximum likelihood for decomposition of recalcitrant substrate.

Table S13 ANOVA table of db-RDA results (a) and variance inflation factors (VIF, b) at the European and country scale. Table S14: Variance partitioning for soil bacterial and fungal communities based on db-RDA.

Table S1
Details of vineyards used for the study. Countries: AT, Austria; CH, Switzerland; DE, Germany; FR, France; RO, Romania; initial state of inter-row treatment before installation of experimental plots: ah, alternating herbicide, at, alternating tillage; cc, complete cover; ti, complete tillage; ch, complete herbicide. Details regarding inter-row treatments are given in Table S2.

country	ID	latitude	longitude	variety	year of planting	initial state of inter-row treatment
AT	AT1	48.4664 °N	15.6612 °E	Pinot Noir	2008	at
	AT2	48.4212 °N	15.6163 °E	Riesling	2007	at
	AT3	47.8139 °N	16.4707 °E	Zweigelt	1988	at
	AT4	47.8621 °N	16.5530 °E	Merlot	2002	at
	AT5	47.8393 °N	16.4901 °E	Carbernet Sauvignon	2001	at
	AT6	47.8399 °N	16.4747 °E	Pinot Noir	2008	at
	AT7[1]	48.4933 °N	15.6448 °E	Green Veltliner	1995	at
	AT8[1]	48.4954 °N	15.6348 °E	Green Veltliner	2000	at
	AT9[1]	48.4849 °N	15.6337 °E	Green Veltliner	2003	at
CH	CH3	46.3201 °N	7.6170 °E	Pinot noir	1984	cc
	CH4	46.3192 °N	7.6185 °E	Pinot noir	1984	ch
	CH6	46.2943 °N	7.5401 °E	Pinot noir	2002	cc
	CH7	46.2790 °N	7.4855 °E	Pinot noir	2005	cc
	CH8	46.2788 °N	7.4856 °E	Pinot noir	1986	ch
	CH9	46.2714 °N	7.4689 °E	Chasselas	2008	cc
	CH10	46.2715 °N	7.4695 °E	Chasselas	1982	ch
	CH11	46.1732 °N	7.1782 °E	Chasselas	1974	cc
	CH12	46.1736 °N	7.1802 °E	Chasselas	1980	ch
	CH13	46.1733 °N	7.1821 °E	Chasselas	1996	ah
	CH14	46.2873 °N	7.5132 °E	Chasselas	2000	ah
	CH15	46.2956 °N	7.5293 °E	Chasselas	1991	cc
	CH16	46.2959 °N	7.5299 °E	Chasselas	1956	ch
	CH17	46.2620 °N	7.5077 °E	Chasselas	2000	ah
	CH18	46.2613 °N	7.5058 °E	Pinot noir	2000	ah
	CH19	46.2995 °N	7.5224°E	Pinot noir	1976	cc
	CH20	46.2976 °N	7.5173 °E	Pinot noir	2006	ah
	CH21	46.2974 °N	7.5157 °E	Pinot noir	2004	ch
	CH22	46.2853 °N	7.5151 °E	Pinot noir	1986	ch
CH	CH23	46.2537 °N	7.4019 °E	Chasselas	2003	cc
	CH24	46.2536 °N	7.4048 °E	Chasselas	2006	ah
	CH25	46.2534 °N	7.4047 °E	Chasselas	1984	ch
	CH26	46.1913 °N	7.2296 °E	Chasselas	1990	ch
	CH27	46.1894 °N	7.2303 °E	Chasselas	1980	cc
	CH28	46.1942 °N	7.2314 °E	Pinot noir	1995	ah
	CH30	46.2529 °N	7.4694 °E	Pinot noir	1985	cc
	CH31	46.2528 °N	7.4683 °E	Pinot noir	1985	ch
	CH32	46.1942 °N	7.2314 °E	Chasselas	1987	ah
	CH33	46.2950 °N	7.5409 °E	Pinot noir	2008	ah
DE	DE1	49.9455°N	7.9949 °E	Pinot noir	2002	cc
	DE2	49.9467 °N	8.0130 °E	Dakapo	2002	cc
	DE3	49.9349 °N	7.9730 °E	Pinot noir	2001	cc

country	ID	latitude	longitude	variety	year of planting	initial state of inter-row treatment
	DE4	49.9254 °N	8.1146 °E	Mueller-Thurgau	1987	cc
	DE5	49.9284 °N	8.1031 °E	Pinot blanc	2007	cc
	DE6	49.8929 °N	8.0765 °E	Silvaner	1988	cc
	DE7	49.8212 °N	8.3080 °E	Dornfelder	1994	cc
	DE8	49.8320 °N	8.3486 °E	Riesling	1994	cc
	DE9	49.8550 °N	8.3545 °E	Not the same across all plots	1992	cc
FR	FR1	44.9484 °N	0.1439 °W	Merlot	1985	cc
	FR2	44.9885 °N	0.0920 °W	Merlot	1995	cc
	FR3	44.9291 °N	0.1641 °W	Merlot	2007	cc
	FR4	44.9471 °N	0.1306 °W	Merlot	2000	cc
	FR5[1]	44.8985 °N	0.1745 °W	Merlot	2000	cc
	FR6	44.8986 °N	0.0332 °W	Merlot	1981	cc
	FR7	44.8580 °N	0.1360 °W	Merlot	1971	cc
	FR8	44.9236 °N	0.0521 °W	Merlot	1983	cc
	FR9	44.8551 °N	0.1582 °W	Merlot	2000	cc
RO	RO1	44.1724 °N	28.3716 °E	Sauvignon blan	2008	ti
	RO2	44.1759 °N	28.4215 °E	Sauvignon blanc	2009	ti
	RO3	44.1814 °N	28.4419 °E	Tămâioasă românească	2009	ti
	RO4	44.0922 °N	27.2984 °E	Cabernet franc	2008	cc
	RO5	44.0943 °N	27.2813 °E	Cabernet franc	2007	cc
RO	RO6	44.1080 °N	27.3047 °E	Cabernet franc	2006	cc
	RO7	44.0806 °N	27.4869 °E	Riesling	2009	cc
	RO8	44.0799 °N	27.5572 °E	Riesling	2006	cc
	RO9	44.0807 °N	27.5743 °E	Riesling	2008	cc

Remarks

[1] Vineyards were organically managed

Table S2

Implementation of vineyard soil disturbance levels “low”, “intermediate”, “high” in the countries involved in the study (AT, Austria; CH, Switzerland; DE, Germany; FR, France; RO, Romania). Denomination, in italics, and description of soil treatments are given.

**soil disturbance**	**AT**	**DE**	**FR**	**RO**	**CH**
low	*complete cover* : inter-row vegetation mulched several times per season on demand				
intermediate	*alternating tillage:* Every second inter-row received only mulching (as in *complete cover*), while the others were treated by mechanical disturbance of the upper soil layer (0-10cm). Tillage was applied 2 – 3 times per season (depending on the region); the inter-rows were switched each season		*green manure* $_{1}$: After grape harvest (beginning of October) vegetation was removed in each inter-row and a winter-crop (*rye-vetch mixture*) was sown. In spring, the winter crop was incorporated into the soil by ploughing	*green manure* $_{2}$ *:* Alfalfa (*Medicago sativa)* was sown in spring, mowed several times over the year as needed and incorporated in the soil in late winter, resown in spring	*alternating herbicide* : Every second inter-row received only mulching (as in *complete cover*), while the others were treated by herbicides (Glyphosate, Glufosinat, Dichlobenil) to remove vegetation; treatment of inter-rows were the same each season.
high	*complete tillage:* In all inter-rows, the upper soil (approx. 0-10cm) was mechanically disturbed. Treatment was applied 2 – 4 times per season (depending on the region)				*complete herbicide* : In all inter-rows, vegetation was removed by herbicide (Glyphosate, Glufosinat, Dichlobenil) application with 1 – 4 applications per growing season starting in April

Table S3

Range, mean values and standard deviation for the included soil parameter covariates (OC [%] = organic carbon; C/N ratio = carbon/nitrogen ratio; Cu [mg/kg] = bioavailable soil copper content; pH = soil pH; clay = clay content in %) at the different disturbance levels (1 = low; 2 = intermediate, 3 = high) in each country (AT, Austria; CH, Switzerland; DE, Germany; FR, France; RO, Romania).

Soil parameters	Country	Range		Disturbance					
				1		2		3	
		min	max	mean	sd	mean	sd	mean	sd
OC [%]	AT	1.69	7.97	3.83	1.64	3.53	1.53	3.43	1.60
	CH	0.95	14.74	4.57	2.36	4.97	3.04	4.04	2.27
	DE	1.53	3.92	2.52	0.52	2.43	0.43	2.41	0.47
	FR	0.75	5.91	1.89	1.10	1.57	0.78	1.68	1.18
	RO	1.29	2.23	1.81	0.26	1.78	0.18	1.82	0.23
C/N ratio	AT	10.3	43.7	20.02	8.41	19.21	7.35	22.07	11.28
	CH	3.6	44	11.81	3.10	13.55	4.52	13.66	6.38
	DE	9.9	18.4	14.06	2.41	13.34	2.02	13.26	2.13
	FR	4.9	17.4	11.57	1.55	11.12	2.01	10.62	2.65
	RO	10.2	15.3	11.76	0.68	11.75	1.38	11.80	0.88
Cu [mg/kg]	AT	1.5	106.1	35.96	30.65	29.31	25.60	29.29	25.47
	CH	15	520.1	79.40	69.75	169.25	172.68	97.32	67.82
	DE	4.1	42.9	24.41	13.26	20.19	10.66	19.58	10.00
	FR	1.3	223.3	54.04	48.36	65.56	57.34	54.52	42.56
	RO	3.6	53.4	17.56	13.81	16.06	12.89	17.60	10.56
pH	AT	6.7	7.6	7.28	0.26	7.31	0.29	7.32	0.25
	CH	5.1	7.7	7.25	0.21	7.12	0.25	7.11	0.65
	DE	7.1	7.6	7.39	0.08	7.39	0.12	7.38	0.11
	FR	5.5	7.4	6.38	0.68	6.48	0.62	6.51	0.61
	RO	6	7.6	7.17	0.55	7.23	0.44	7.20	0.49
Clay %	AT	6	32	16.83	7.41	17.22	7.90	18.06	6.95
	CH	4	18	9.10	3.96	8.48	3.17	11.33	3.90
	DE	0	37	17.78	9.04	15.78	6.43	14.44	5.94
	FR	4	22	10.33	6.23	11.22	3.90	11.89	5.30
	RO	14	26	21.44	3.91	21.33	4.28	21.78	3.72

Table S4

Summary of general linear mixed model fit by maximum likelihood for the response of each soil covariate (OC [%] = organic carbon; C/N ratio = carbon/nitrogen ratio; Cu [mg/kg] = bioavailable soil copper content; pH = soil pH; clay = clay content in %) to the disturbance gradient (low = 1; intermediate = 2; high = 3). t-tests used Satterthwaite's method ['lmerModLmerTest'].

Response variable	Explanatory variable	Estimate	Std. Error	df	t value	Pr(>|t|)
OC %		-0.046	0.03	187.996	-1.541	0.125
C/N ratio	~ disturbance (1 - 3)	0.005	0.021	186	0.218	0.828
Cu [mg/kg]		-0.004	0.091	132.017	-0.043	0.966
pH		-0.008	0.034	186.074	-0.242	0.809
clay %		0.207	0.602	132.006	0.343	0.732

Table S5

Summary of coefficients from linear mixed effect model fitted by maximum likelihood for bacterial OTU richness. Plot within vineyard was included in the models as nested random factor (1|vineyard/ plot/year) and country (AT, Austria; CH, Switzerland; DE, Germany; FR, France; RO, Romania) was included as interaction term for all analyses. The symbol ":" in the fixed factor collumn, indicates the interaction of the fixed factor with the country. (Significances were obtained by t-tests using Satterthwaite's method; '***' significant at p < 0.001; '**' significant at p < 0.01; '*' significant at p < 0.05; '.' significant at p < 0.1)

fixed factor	Estimate	Std. Error	df	t value	Pr(>|t|)	sig
(Intercept)	2023.66	116.12	259.64	17.43	0	***
disturbance	15.19	52.53	254.61	0.29	0.773	
Cu	33.32	40.78	368.54	0.82	0.414	
C org	-159.5	64.76	356.34	-2.46	0.014	*
C/N	29.08	44.61	352.54	0.65	0.515	
pH	-40.84	106.73	340.21	-0.38	0.702	
clay	57.92	42.6	358.37	1.36	0.175	
countryFR	-937.98	141.53	346.82	-6.63	0	***
countryDE	-119.28	123.7	342.99	-0.96	0.336	
countryRO	-871.28	198.48	323.22	-4.39	0	***
countryCH	-337.11	129.5	381.74	-2.6	0.01	**
disturbance : countryFR	77.95	61.89	346.98	1.26	0.209	
disturbance : countryDE	18.35	45.58	306.69	0.4	0.688	
disturbance : countryRO	-1.34	60.27	350.43	-0.02	0.982	
disturbance : countryCH	-4.74	55.92	397.76	-0.08	0.933	
Cu : countryFR	-91.68	58.26	346.06	-1.57	0.116	
Cu : countryDE	-141.77	63.79	389.5	-2.22	0.027	*
Cu : countryRO	-66.4	76.12	330.71	-0.87	0.384	
Cu : countryCH	-68.12	58.13	382.28	-1.17	0.242	
C org : countryFR	233.68	91.47	325.39	2.55	0.011	*
C org : countryDE	216.9	102	351.25	2.13	0.034	*
C org : countryRO	432.39	152.94	334.55	2.83	0.005	**
C org : countryCH	261.06	73.21	376.48	3.57	0	***
C/N : countryFR	-62.98	67.55	326.14	-0.93	0.352	
C/N : countryDE	47.81	68.49	366.35	0.7	0.486	
C/N : countryRO	-260.3	157.88	327.72	-1.65	0.1	
C/N : countryCH	-104.64	53.8	368.05	-1.94	0.053	.
pH : countryFR	-21.39	112.05	347.79	-0.19	0.849	
pH : countryDE	190.63	158.92	345.02	1.2	0.231	
pH : countryRO	91.46	114.54	335.69	0.8	0.425	
pH : countryCH	96.32	115.08	385.43	0.84	0.403	
clay : countryFR	-134.41	79.01	346.46	-1.7	0.09	.
clay : countryDE	-63.71	51.59	363.96	-1.23	0.218	
clay : countryRO	96.55	86.25	344.44	1.12	0.264	
clay : countryCH	-108.46	72.85	346.91	-1.49	0.137	

Table S6

Summary coefficients from linear mixed effect model fitted by maximum likelihood for bacterial OTU shannon index. Plot within vineyard was included in the models as nested random factor (1|vineyard/plot/year) and country (AT, Austria; CH, Switzerland; DE, Germany; FR, France; RO, Romania) was included as interaction term for all analyses. The symbol ":" in the fixed factor collumn, indicates the interaction of the fixed factor with the country. (Significances were obtained by t-tests using Satterthwaite's method; '***' significant at p < 0.001; '**' significant at p < 0.01; '*' significant at p < 0.05; '.' significant at p < 0.1)

Fixed Factor	Estimate	Std. Error	df	t value	Pr(>|t|)	sig
(Intercept)	6.316116	0.069146	264.1551	91.3442	<0.001	***
disturbance	0.023887	0.030576	215.8338	0.781218	0.436	
Cu	0.048545	0.026807	381.5011	1.810866	0.071	
OC	-0.09759	0.043048	354.9685	-2.26706	0.024	*
C/N	0.013541	0.029605	365.9419	0.457403	0.648	
pH	-0.07909	0.071257	349.9693	-1.10995	0.268	
clay	0.027148	0.028259	362.8586	0.960711	0.337	
CountryFR	-0.2996	0.092634	381.3007	-3.23427	<0.001	***
CountryDE	0.209274	0.082128	362.5173	2.548155	0.011	*
CountryRO	-0.12271	0.131826	356.5133	-0.93084	0.353	
CountryCH	0.11595	0.084331	382.2085	1.37495	0.170	
disturbance : CountryFR	0.044835	0.040355	374.5771	1.111026	0.267	
disturbance : CountryDE	-0.01487	0.030514	320.4614	-0.48728	0.626	
disturbance : CountryRO	-0.01668	0.039214	375.621	-0.42526	0.671	
disturbance : CountryCH	-0.00917	0.035709	379.3453	-0.25678	0.797	
Cu : CountryFR	-0.07089	0.038818	354.3887	-1.82631	0.069	.
Cu : CountryDE	-0.11761	0.042234	284.7759	-2.78475	0.006	**
Cu : CountryRO	-0.08108	0.050733	355.8854	-1.59822	0.111	
Cu : CountryCH	-0.14888	0.03699	281.7338	-4.02502	<0.001	***
OC : CountryFR	0.157312	0.060851	354.5405	2.585195	0.010	*
OC : CountryDE	0.107011	0.067591	369.4361	1.583201	0.114	
OC: CountryRO	0.324323	0.10282	324.617	3.154276	0.002	**
OC : CountryCH	0.181851	0.048205	357.3553	3.772468	<0.001	***
C/N : CountryFR	-0.01839	0.045213	342.4341	-0.40675	0.684	
C/N : CountryDE	0.01648	0.045262	366.4356	0.364099	0.716	
C/N : CountryRO	-0.23686	0.105041	357.2218	-2.25492	0.025	*
C/N : CountryCH	-0.06592	0.035336	381.2228	-1.8656	0.063	.
pH : CountryFR	0.021085	0.074653	354.2858	0.282445	0.778	
pH : CountryDE	0.196823	0.105291	369.673	1.869317	0.062	.
pH : CountryRO	0.164421	0.076248	359.9146	2.156382	0.032	*
pH : CountryCH	0.095831	0.075491	364.9807	1.269441	0.205	
clay : CountryFR	-0.12925	0.052756	345.6988	-2.44988	0.015	*
clay : CountryDE	-0.04043	0.034276	347.853	-1.17961	0.239	
clay : CountryRO	0.066003	0.057632	342.2233	1.145259	0.253	
clay : CountryCH	-0.06362	0.045582	248.7779	-1.39573	0.164	

Table S7

Summary coefficients from linear mixed effect model fitted by maximum likelihood for fungal OTU richness. Plot within vineyard was included in the models as nested random factor (1|vineyard/ plot/year) and country (AT, Austria; CH, Switzerland; DE, Germany; FR, France; RO, Romania) was included as interaction term for all analyses. The symbol ":" in the fixed factor collumn, indicates the interaction of the fixed factor with the country. (Significances were obtained by t-tests using Satterthwaite's method; '***' significant at p < 0.001; '**' significant at p < 0.01; '*' significant at p < 0.05; '.' significant at p < 0.1)

Fixed factor	Estimate	Std. Error	df	t value	Pr(>|t|)	sig
(Intercept)	386.46	35.49	253.68	10.89	0	***
disturbance	-32.95	16.1	250.64	-2.05	0.042	*
Cu	28.15	11.71	358.44	2.4	0.017	*
C org	-37.98	18.44	349.47	-2.06	0.04	*
C/N	9.91	12.73	347.78	0.78	0.437	
pH	-83.51	30.21	337.42	-2.76	0.006	**
clay	44.6	12.16	352.35	3.67	0	***
CountryFR	-186.4	40.59	343.28	-4.59	0	***
CountryDE	26.45	35.43	341.1	0.75	0.456	
CountryRO	-349.76	56.79	324.23	-6.16	0	***
CountryCH	-120.13	36.5	375.29	-3.29	0.001	**
disturbance : CountryFR	49.71	17.69	342.32	2.81	0.005	**
disturbance : CountryDE	48.81	12.9	313.81	3.78	0	***
disturbance : CountryRO	64.24	17.32	344.27	3.71	0	***
disturbance : CountryCH	13.34	16.15	397.52	0.83	0.409	
Cu : CountryFR	-31.86	16.67	342.63	-1.91	0.057	.
Cu : CountryDE	-19.33	18.55	378.69	-1.04	0.298	
Cu : CountryRO	-50.01	21.89	331.56	-2.28	0.023	*
Cu : CountryCH	-25.91	17.11	392.48	-1.51	0.131	
C org : CountryFR	56.71	25.91	325.87	2.19	0.029	*
C org : CountryDE	47.99	29.26	344.99	1.64	0.102	
C org : CountryRO	36.03	43.44	334.12	0.83	0.408	
C org : CountryCH	65.78	20.81	364.1	3.16	0.002	**
C/N : CountryFR	-7.65	19.19	327.65	-0.4	0.69	
C/N : CountryDE	14.05	19.63	359.37	0.72	0.475	
C/N : CountryRO	-15.92	44.89	329.19	-0.35	0.723	
C/N : CountryCH	-34.02	15.3	356.51	-2.22	0.027	*
pH : CountryFR	60.63	31.79	343.9	1.91	0.057	.
pH : CountryDE	102.39	45.35	341.6	2.26	0.025	*
pH : CountryRO	64.27	32.44	333.92	1.98	0.048	*
pH : CountryCH	103.02	33.05	388.63	3.12	0.002	**
clay : CountryFR	-36.92	22.56	343.36	-1.64	0.103	
clay : CountryDE	-41.62	14.77	355.63	-2.82	0.005	**
clay : CountryRO	-27.35	24.61	340.44	-1.11	0.267	
clay : CountryCH	-34.89	21.44	368.53	-1.63	0.104	

Table S8

Summary coefficients from linear mixed effect model fitted by maximum likelihood for fungal OTU shannon index. Plot within vineyard was included in the models as nested random factor (1|vineyard/ plot/year) and country (AT, Austria; CH, Switzerland; DE, Germany; FR, France; RO, Romania) was included as interaction term for all analyses. The symbol ":" in the fixed factor collumn, indicates the interaction of the fixed factor with the country. (Significances were obtained by t-tests using Satterthwaite's method; '***' significant at p < 0.001; '**' significant at p < 0.01; '*' significant at p < 0.05; '.' significant at p < 0.1)

Fixed factor	Estimate	Std. Error	df	t value	Pr(>|t|)	sig
(Intercept)	4.191	0.177	298.67	23.624	<0.001	***
disturbance	0.009	0.080	292.91	0.109	0.913	
Cu	0.097	0.071	388.46	1.371	0.171	
C org	0.004	0.114	400.85	0.037	0.970	
C/N	0.080	0.079	401.00	1.017	0.310	
pH	-0.454	0.190	398.52	-2.388	0.017	*
clay	0.046	0.075	399.68	0.618	0.537	
CountryFR	-1.115	0.251	399.79	-4.433	<0.001	***
CountryDE	0.097	0.224	391.75	0.433	0.665	
CountryRO	-1.227	0.364	392.79	-3.376	<0.001	***
CountryCH	-0.327	0.220	399.23	-1.490	0.137	
disturbance : CountryFR	-0.099	0.110	400.40	-0.903	0.367	
disturbance : CountryDE	-0.058	0.085	351.29	-0.687	0.493	
disturbance : CountryRO	0.022	0.107	399.88	0.208	0.835	
disturbance : CountryCH	-0.147	0.093	381.65	-1.583	0.114	
Cu : CountryFR	-0.090	0.104	400.41	-0.861	0.390	
Cu : CountryDE	-0.071	0.108	357.96	-0.658	0.511	
Cu : CountryRO	-0.279	0.139	398.34	-2.013	0.045	*
Cu : CountryCH	-0.119	0.094	337.72	-1.268	0.206	
C org : CountryFR	-0.157	0.166	391.35	-0.948	0.344	
C org : CountryDE	-0.108	0.182	400.99	-0.591	0.555	
C org : CountryRO	0.094	0.276	391.74	0.342	0.733	
C org : CountryCH	0.070	0.126	396.76	0.552	0.581	
C/N : CountryFR	0.082	0.123	392.61	0.670	0.503	
C/N : CountryDE	-0.043	0.120	398.84	-0.359	0.720	
C/N : CountryRO	0.183	0.286	393.52	0.639	0.523	
C/N : CountryCH	-0.115	0.094	399.82	-1.228	0.220	
pH : CountryFR	0.273	0.199	400.18	1.372	0.171	
pH : CountryDE	0.578	0.283	400.33	2.038	0.042	*
pH : CountryRO	0.289	0.205	397.45	1.406	0.160	
pH : CountryCH	0.441	0.199	400.87	2.212	0.028	*
clay : CountryFR	0.224	0.141	400.70	1.591	0.112	
clay : CountryDE	-0.008	0.090	396.16	-0.093	0.926	
clay : CountryRO	0.283	0.154	400.85	1.838	0.067	.
clay : CountryCH	0.029	0.113	307.65	0.259	0.796	

Table S9

Results of Indicator species analysis of bacterial and fungal communities at European and country level (AT, Austria; CH, Switzerland; DE, Germany; FR, France; RO, Romania). Values for specitivity (Value A) and sensitivity (value B) and indicator values (Ind. Val.)follow De Cáceres, Legendre, 2009. P-values based on permutation tests (‘***’ significant at p < 0.001; ‘**’ p < 0.01; ‘*’ p < 0.05; ‘.’ p < 0.1). Relative abundances correspond to total sequence numbers in the given data set. Indicator taxa for individual or combined levels of soil disturbance are shown, if they met the pre-defined criteria (Ind. Value > 0.6, p < 0.05). Indicator taxa for the European level or for at least two countries are given in bold.

Taxon	Data set	A	B	Ind. Val.	p-value		relative abundance [%]
**bacteria**							
**soil disturbance 'low'**							
Aquabacterium	AT	0.9516	0.4444	0.6503007	0.0044	**	0.004
Actinocorallia	CH	0.7755	0.475	0.6069287	0.0002	***	0.006
Bauldia	FR	0.7778	0.5556	0.6573779	0.0011	**	0.005
Demequinaceae_unclassified	FR	0.6582	0.5556	0.604728	0.0058	**	0.006
Gp1_unclassified	RO	0.5974	0.6667	0.6310995	0.0465	*	0.022
**soil disturbance 'low + intermediate'**							
**Dyadobacter**	AT	0.8571	0.8333	0.8451162	0.0012	**	0.021
Enterobacteriaceae_unclassified	AT	0.978	0.6389	0.7904709	0.0054	**	0.022
Acinetobacter	AT	0.9913	0.3889	0.6208998	0.0112	*	0.007
**Dyadobacter**	CH	0.8954	0.7111	0.7979467	0.0001	***	0.017
Tahibacter	CH	0.8858	0.5222	0.6801211	0.0013	**	0.012
Sphingopyxis	CH	0.8822	0.5	0.6641536	0.0014	**	0.007
Taibaiella	CH	0.9607	0.3889	0.6112415	0.0008	***	0.006
Smaragdicoccus	DE	0.8904	0.4167	0.6091221	0.0055	**	0.003
Epilithonimonas	FR	1	0.25	0.5	0.049	*	0.003
**soil disturbance 'low + high'**							
Peredibacter	RO	0.8706	0.6111	0.7293995	0.0369	*	0.012
Desulfuromonas	FR	0.946	0.5833	0.7428336	0.0039	**	0.020
**soil disturbance 'intermediate'**							
Nannocystis	FR	0.8809	0.6667	0.7663524	0.0126	*	0.030

Taxon	Data set	A	B	Ind. Val.	p-value		relative abundance [%]
Enterobacter	FR	0.7747	0.7222	0.7479895	0.0452	*	0.056
**soil disturbance 'intermediate + high'**							
**Pontibacter**	Europe	0.90777	0.58736	0.7301971	0.0001	***	0.041
**Hymenobacter**	Europe	0.91527	0.52788	0.6950919	0.0001	***	0.016
**Pontibacter**	AT	0.8755	1	0.9356816	0.0001	***	0.059
**Hymenobacter**	AT	0.9545	0.8333	0.8918435	0.0002	**	0.029
Cytophagaceae_unclassified	AT	0.8509	0.8889	0.8696925	0.001	**	0.025
**Armatimonadetes_unclassified**	AT	0.9054	0.75	0.8240449	0.0007	***	0.011
Beijerinckiaceae_unclassified	AT	0.886	0.7222	0.7999182	0.0018	**	0.008
Byssovorax	AT	0.8516	0.75	0.7991871	0.0103	*	0.008
Nocardiopsis	AT	0.9211	0.5556	0.7153762	0.0109	*	0.005
Segetibacter	AT	0.9694	0.5	0.696204	0.0094	**	0.006
Chloroflexales_unclassified	AT	0.8571	0.5278	0.6725901	0.0408	*	0.014
Acidobacteria_Gp1_unclassified	CH	0.9525	0.6067	0.7601853	0.002	**	0.037
**Pontibacter**	DE	0.9573	0.9583	0.9577999	0.0001	***	0.086
Alcaligenaceae_unclassified	DE	0.9007	0.8194	0.8590888	0.0001	***	0.022
**Hymenobacter**	DE	0.946	0.6944	0.8104952	0.0001	***	0.021
Rhodocytophaga	DE	0.897	0.7222	0.8048686	0.0001	***	0.012
Kaistia	DE	0.8884	0.6389	0.7533915	0.0004	***	0.009
Hydrogenophaga	DE	0.8994	0.5694	0.7156245	0.0002	***	0.007
Algoriphagus	DE	0.8828	0.5417	0.6915293	0.0016	**	0.009
Flavobacteriaceae_unclassified	DE	0.8607	0.5556	0.6915236	0.0013	**	0.005
**Armatimonadetes_unclassified**	DE	0.8298	0.4583	0.6166825	0.0048	**	0.003
Massilia	FR	0.9477	0.8889	0.9178292	0.0001	***	0.080
Sorangium	FR	0.9251	0.8611	0.8925265	0.0001	***	0.049
Variovorax	FR	0.8897	0.8889	0.8892999	0.0006	***	0.039
Herpetosiphon	FR	0.8897	0.8056	0.8466064	0.0009	***	0.023
Peredibacter	FR	0.9371	0.75	0.8383466	0.0059	**	0.026
Geodermatophilus	FR	0.8547	0.8056	0.8297869	0.0005	***	0.030
Myxococcus	FR	0.8806	0.7222	0.7974768	0.0058	**	0.020
Chitinophaga	FR	0.98	0.6389	0.7912787	0.0001	***	0.033

Taxon	Data set	A	B	Ind. Val.	p-value		relative abundance [%]
Armatimonadetes_unclassified	FR	0.9176	0.6667	0.7821534	0.0003	***	0.013
Cystobacter	FR	0.931	0.6389	0.7712431	0.0006	***	0.019
Achromobacter	FR	0.9815	0.5556	0.7384588	0.0042	**	0.016
Porphyrobacter	FR	0.9465	0.5	0.6879317	0.0066	**	0.014
**Pontibacter**	FR	0.8833	0.5278	0.6827926	0.0054	**	0.026
Sphingobium	FR	0.9512	0.4722	0.6701915	0.0276	*	0.018
**Hymenobacter**	FR	0.9776	0.4444	0.6591248	0.0145	*	0.016
Rubellimicrobium	FR	0.92	0.4722	0.6591085	0.0174	*	0.009
**soil disturbance 'high'**							
Parasegetibacter	DE	0.7667	0.5278	0.6361323	0.0001	***	> 0.001
**fungi**							
**soil disturbance 'low'**							
s__Cistella_albidolutea_SH198116.07FU	AT	0.8676	0.5556	0.6942473	0.0008	***	0.212
s__Phaeosphaeria_sp_SH182986.07FU	CH	0.94561	0.40476	0.6186668	0.0002	***	0.123
f__Claroideoglomeraceae	DE	0.8857	0.6571	0.762893	0.0001	***	0.023
g__Claroideoglomus	DE	0.8197	0.4571	0.6121499	0.0002	***	0.022
f__Tubeufiaceae	FR	0.9749	1	0.9873712	0.0001	***	0.044
s__Pyrenochaeta_sp_SH019261.07FU	FR	0.9974	0.94444	0.9705532	0.0001	***	0.117
g__Cyphellophora	FR	0.8617	0.7778	0.8186524	0.0002	***	0.027
s__Lecanicillium_primulinum_SH204659.07FU	FR	0.9675	0.6667	0.8031278	0.0001	***	0.020
s__Scytalidium_circinatum_SH218618.07FU	FR	0.9643	0.6667	0.8017837	0.0001	***	0.011
s__Heteroconium_sp_SH198136.07FU	FR	0.8693	0.7222	0.7923611	0.0005	***	0.029
g__Lecanicillium	FR	0.9922	0.6111	0.7787001	0.0001	***	0.004
s__Pyrenochaetopsis_pratorum_SH198998.07FU	FR	0.9547	0.6111	0.7638332	0.0001	***	0.053
s__Cylindrotrichum_sp_SH456111.07FU	FR	0.9442	0.6111	0.7596069	0.0001	***	0.015
s__Tetracladium_sp_SH026253.07FU	FR	0.9102	0.6111	0.7458022	0.0002	***	0.011
s__Auricularia_sp_SH024175.07FU	FR	1	0.05556	0.745356	0.0001	***	0.017
s__Cryptococcus_sp_SH181630.07FU	FR	0.7561	0.6667	0.7099754	0.0177	*	0.004
s__Phoma_paspali_SH215817.07FU	FR	0.9591	0.5	0.6925056	0.0004	***	0.025

Taxon	Data set	A	B	Ind. Val.	p-value	relative abundance [%]
g__Schwanniomyces	FR	0.956	0.4444	0.6518234	0.0059 **	0.048
s__Colletotrichum_destructivum_SH375584.07FU	FR	0.8045	0.5	0.6342497	0.0025 **	0.008
f__Gnomoniaceae	FR	0.9967	0.3889	0.6225869	0.014 *	0.073
f__Hypocreaceae	FR	0.8721	0.4444	0.6225728	0.0081 **	0.003
g__Colletotrichum	FR	0.7304	0.5	0.6043146	0.0107 *	0.007
**soil disturbance 'low + intermediate'**						
**f__Glomeraceae**	AT	0.9166	0.5278	0.6955305	0.0077 **	0.057
s__Cryptococcus_chernovii_SH197624.07FU	AT	0.947	0.4722	0.668736	0.0123 *	0.037
f__Pleosporales_fam_Incertae_sedis	CH	0.9679	0.5158	0.7065686	0.0029 **	0.319
s__Monographella_cucumerina_SH527845.07FU	CH	0.967	0.4526	0.6615904	0.0026 **	0.346
**f__Glomeraceae**	DE	0.9263	0.8429	0.8835932	0.0001 ****	0.245
**s__Sarocladium_strictum_SH210203.07FU**	DE	0.9138	0.4286	0.6257871	0.0165 *	0.474
s__Pyrenochaetopsis_leptospora_SH198996.07FU	FR	0.9714	1	0.985592	0.0001 ***	0.474
s__Cryptococcus_heimaeyensis_SH181631.07FU	FR	0.8512	0.6111	0.7212177	0.0187 *	0.007
**s__Sarocladium_strictum_SH210203.07FU**	FR	0.9153	0.5278	0.6950186	0.0108 *	0.008
**soil disturbance 'low + high'**						
s__Tetracladium_sp_SH211298.07FU	CH	0.9674	0.4634	0.6695681	0.0071 **	0.153
g__Preussia	CH	0.925	0.4512	0.6460593	0.0003 ***	0.269
g__Tetracladium	CH	0.8628	0.4512	0.6239486	0.0059 **	0.067
s__Mycoarthris_corallina_SH198115.07FU	CH	0.9025	0.4146	0.6117204	0.0181 *	0.351
o__Agaricales	FR	0.9798	0.9444	0.9619799	0.0212 *	0.713
g__Ilyonectria	FR	0.9859	0.7778	0.8756933	0.0495 *	0.192
s__Pyrenochaeta_sp_SH014374.07FU	FR	0.974	0.7778	0.8546818	0.0001 ***	0.121
s__Cyphellophora_vermispora_SH105839.07FU	FR	0.974	0.75	0.8528954	0.0017 **	0.046
s__Dactylaria_dimorphospora_SH025283.07FU	FR	0.9344	0.5556	0.7204962	0.026 *	0.057
g__Conocybe	FR	0.9771	0.4444	0.6590036	0.0046 **	0.006
s__Acremonium_rutilum_SH207064.07FU	FR	0.9578	0.4444	0.6524457	0.0053 **	0.011
**soil disturbance 'intermediate**						

Taxon	Data set	A	B	Ind. Val.	p-value		relative abundance [%]
s__Stemphylium_herbarum_SH387143.07FU	RO	0.8617	0.5556	0.6918791	0.0005	***	0.005
**soil disturbance 'intermediate + high'**							
f__Pleosporales_fam_Incertae_sedis	AT	0.8874	0.75	0.8158327	0.0048	**	0.200
s__Mortierella_antarctica_SH220322.07FU	AT	0.9452	0.4167	0.6275514	0.0213	*	0.018
c__Chytridiomycetes	DE	0.8317	0.7606	0.7953596	0.0004	***	0.038
**s__Spizellomyces_dolichospermus_SH183868.07FU**	DE	0.8962	0.7042	0.7944132	0.0001	***	0.073
s__Minimedusa_polyspora_SH201127.07FU	FR	0.9513	0.7222	0.8288733	0.0012	**	0.031
s__Cladorrhinum_sp_SH522900.07FU	FR	0.9	0.6944	0.803641	0.004	**	0.042
p__Chytridiomycota	FR	0.8152	0.75	0.7819411	0.0018	**	0.015
g__Spizellomyces	FR	0.9868	0.5556	0.7404361	0.0011	**	0.013
**s__Spizellomyces_dolichospermus_SH183868.07FU**	FR	0.9116	0.5833	0.7292314	0.0118	*	0.021
g__Cladorrhinum	FR	0.9761	0.5278	0.7177526	0.0072	**	0.010
s__Mortierella_sp_SH216193.07FU	FR	0.9833	0.4722	0.6814176	0.0012	**	0.012
g__Helgardia	FR	0.9042	0.4722	0.6534248	0.014	*	0.019
s__Mortierella_sp_SH180140.07FU	FR	0.8864	0.4444	0.6276459	0.0019	**	0.006
**soil disturbance 'high'**							
s__Spizellomyces_pseudodichotomus_SH196353.07FU	AT	0.9035	0.6111	0.7430757	0.0001	***	0.0725329
c__Chytridiomycetes	AT	0.7844	0.5	0.6262617	0.0006	***	0.0586327
s__Cryptococcus_adeliensis_SH405379.07FU	AT	0.8377	0.4444	0.6101579	0.0343	*	0.0772907
s__Hymenoscyphus_sp_SH174399.07FU	FR	0.9931	0.5556	0.7427956	0.0491	*	0.6720102

Table S10

Coefficients from linear mixed effect model fitted by maximum likelihood for microbial respiration. Plot within vineyard was included in the models as nested random factor (1|vineyard/ plot/year) and country (AT, Austria; CH, Switzerland; DE, Germany; FR, France; RO, Romania) was included as interaction term for all analyses. The symbol ":" in the fixed factor collumn, indicates the interaction of the fixed factor with the country. (Significances were obtained by t-tests using Satterthwaite's method; '***' significant at $p < 0.001$; '**' significant at $p < 0.01$; '*' significant at $p < 0.05$; '.' significant at $p < 0.1$)

Fixed Factor	Estimate	Std. Error	df	t value	Pr(>|t|)	sig
(Intercept)	1.458	0.065	162.05	22.56	0	***
disturbance	-0.109	0.026	93.72	-4.25	0	***
Cu	-0.019	0.024	577.39	-0.8	0.423	
C org	0.26	0.041	507.36	6.42	0	***
C/N	-0.142	0.035	600.37	-4.09	0	***
pH	-0.086	0.073	573.32	-1.18	0.24	
clay	-0.028	0.026	545.19	-1.07	0.284	
CountryFR	-0.27	0.075	609.48	-3.6	0	***
CountryDE	-0.37	0.126	616.63	-2.94	0.003	**
CountryRO	-0.204	0.109	624.59	-1.87	0.062	.
CountryCH	-0.055	0.1	318.76	-0.55	0.582	
disturbance : CountryFR	0.023	0.03	570.59	0.77	0.442	
disturbance : CountryDE	0.181	0.035	569.8	5.2	0	***
disturbance : CountryRO	0.054	0.033	561.05	1.64	0.101	
disturbance : CountryCH	-0.056	0.038	270.3	-1.49	0.137	
Cu : CountryFR	-0.101	0.034	625.26	-2.97	0.003	**
Cu : CountryDE	0.042	0.055	559.81	0.77	0.44	
Cu : CountryRO	-0.016	0.047	628.1	-0.35	0.729	
Cu : CountryCH	0.121	0.04	139.59	3.02	0.003	**
C org : CountryFR	0.005	0.058	618.39	0.09	0.929	
C org : CountryDE	0.185	0.084	616.94	2.2	0.028	*
C org : CountryRO	-0.189	0.095	616.06	-2	0.046	*
C org : CountryCH	-0.007	0.05	360.25	-0.14	0.886	
C/N : CountryFR	0.069	0.04	624.64	1.74	0.083	.
C/N : CountryDE	-0.039	0.079	574.24	-0.5	0.62	
C/N : CountryRO	0.135	0.047	622.53	2.9	0.004	**
C/N : CountryCH	0.084	0.051	482.82	1.67	0.096	.
pH : CountryFR	0.037	0.077	570.23	0.48	0.63	
pH : CountryDE	-0.059	0.153	627.48	-0.39	0.7	
pH : CountryRO	0.046	0.076	627.08	0.6	0.548	
pH : CountryCH	0.139	0.083	327.7	1.67	0.097	.
clay : CountryFR	-0.079	0.055	420.76	-1.45	0.148	
clay : CountryDE	0.052	0.045	606.23	1.15	0.253	
clay : CountryRO	0.137	0.057	430.43	2.41	0.017	*
clay : CountryCH	0.11	0.048	116.34	2.3	0.023	*

Table S11

Summary coefficients from linear mixed effect model fitted by maximum likelihood for decomposition of labile substrate. Plot within vineyard was included in the models as nested random factor (1|vineyard/ plot/year) and country (AT, Austria; CH, Switzerland; DE, Germany; FR, France; RO, Romania) was included as interaction term for all analyses. The symbol ":" in the fixed factor collumn, indicates the interaction of the fixed factor with the country. (Significances were obtained by t-tests using Satterthwaite's method; '***' significant at $p < 0.001$; '**' significant at $p < 0.01$; '*' significant at $p < 0.05$; '.' significant at $p < 0.1$)

Fixed factor	Estimate	Std. Error	df	t value	Pr(>|t|)	sig
(Intercept)	52.794	1.832	216.3	28.82	0	***
disturbance	0.718	0.741	187.74	0.97	0.334	
Cu	-0.39	0.757	311.12	-0.52	0.607	
C org	0.721	1.235	306.5	0.58	0.56	
C/N	-1.909	1.02	310.21	-1.87	0.062	.
pH	0.939	2.105	298.4	0.45	0.656	
clay	-0.345	0.81	299.87	-0.43	0.671	
CountryFR	3.367	2.384	287.41	1.41	0.159	
CountryDE	10.729	3.562	284.09	3.01	0.003	**
CountryRO	5.378	3.47	289.88	1.55	0.122	
CountryCH	1.297	2.822	267.22	0.46	0.646	
disturbance : CountryFR	1.807	0.985	256.83	1.83	0.068	.
disturbance : CountryDE	-2.801	1.06	245.49	-2.64	0.009	**
disturbance : CountryRO	-0.116	1.025	248.13	-0.11	0.91	
disturbance : CountryCH	-0.748	1.111	252.39	-0.67	0.501	
Cu : CountryFR	-1.443	1.03	285.09	-1.4	0.162	
Cu : CountryDE	1.598	1.527	305.4	1.05	0.296	
Cu : CountryRO	1.864	1.465	310.07	1.27	0.204	
Cu : CountryCH	4.179	1.255	126.36	3.33	0.001	**
C org : CountryF	-0.935	1.555	266.85	-0.6	0.548	
C org : CountryDE	-2.495	2.594	305.2	-0.96	0.337	
C org : CountryRO	-5.947	3.048	304.5	-1.95	0.052	
C org : CountryCH	0.137	1.433	286.59	0.1	0.924	
C/N : CountryF	3.605	1.169	285.71	3.08	0.002	**
C/N : CountryDE	2.985	2.089	306.56	1.43	0.154	
C/N : CountryRO	10.582	3.543	257.21	2.99	0.003	**
C/N : CountryCH	-0.23	1.438	277.64	-0.16	0.873	
pH : CountryFR	0.075	2.216	306.03	0.03	0.973	
pH : CountryDE	4.68	4.276	287.01	1.09	0.275	
pH : CountryRO	-2.837	2.202	289.69	-1.29	0.199	
pH : CountryCH	-0.88	2.352	295.15	-0.37	0.708	
clay : CountryFR	-1.189	1.335	288.25	-0.89	0.374	
clay : CountryDE	1.36	1.133	298.87	1.2	0.231	
clay : CountryRO	5.559	1.712	306.7	3.25	0.001	**
clay : CountryCH	1.188	1.456	141.6	0.82	0.416	

Table S12

Summary coefficients from linear mixed effect model fitted by maximum likelihood for decomposition of recalcitrant substrate. Plot within vineyard was included in the models as nested random factor (1|vineyard/ plot/year) and country (AT, Austria; CH, Switzerland; DE, Germany; FR, France; RO, Romania) was included as interaction term for all analyses. The symbol ":" in the fixed factor collumn, indicates the interaction of the fixed factor with the country. (Significances were obtained by t-tests using Satterthwaite's method; '***' significant at p < 0.001; '**' significant at p < 0.01; '*' significant at p < 0.05; '.' significant at p < 0.1)

Fixed factor	Estimate	Std. Error	df	t value	Pr(>|t|)	sig
(Intercept)	24.923	2.124	301	11.74	0	***
disturbance	2.399	0.934	301	2.57	0.011	*
Cu	0.391	0.854	301	0.46	0.647	
C org	0.719	1.39	301	0.52	0.605	
C/N	0.071	1.18	301	0.06	0.952	
pH	-0.101	2.452	301	-0.04	0.967	
clay	-2.124	0.918	301	-2.31	0.021	*
CountryFR	32.52	3.029	301	10.74	0	***
CountryDE	10.918	4.338	301	2.52	0.012	*
CountryRO	-12.643	4.272	301	-2.96	0.003	**
CountryCH	-0.09	3.306	301	-0.03	0.978	
disturbance : CountryFR	-1.707	1.31	301	-1.3	0.194	
disturbance : CountryDE	-3.107	1.346	301	-2.31	0.022	*
disturbance : CountryRO	-0.527	1.309	301	-0.4	0.687	
disturbance : CountryCH	-0.006	1.304	301	0	0.997	
Cu : CountryFR	-1.545	1.262	301	-1.22	0.222	
Cu : CountryDE	0.833	1.686	301	0.49	0.622	
Cu : CountryRO	1.012	1.725	301	0.59	0.558	
Cu : CountryCH	-2.332	1.344	301	-1.73	0.084	.
C org : CountryFR	-0.079	1.978	301	-0.04	0.968	
C org : CountryDE	-1.991	3.029	301	-0.66	0.511	
C org : CountryRO	1.256	3.454	301	0.36	0.716	
C org : CountryCH	1.16	1.601	301	0.72	0.469	
C/N : CountryFR	-0.177	1.418	301	-0.12	0.901	
C/N : CountryDE	2.669	2.384	301	1.12	0.264	
C/N : CountryRO	3.149	4.357	301	0.72	0.47	
C/N : CountryCH	3.576	1.652	301	2.17	0.031	*
pH : CountryFR	0.205	2.57	301	0.08	0.936	
pH : CountryDE	0.98	5.172	301	0.19	0.85	
pH : CountryRO	-2.289	2.644	301	-0.87	0.387	
pH : CountryCH	0.826	2.707	301	0.31	0.761	
clay : CountryFR	1.331	1.537	301	0.87	0.387	
clay : CountryDE	3.302	1.271	301	2.6	0.01	**
clay : CountryRO	11.184	1.896	301	5.9	0	***
clay : CountryCH	1.667	1.582	301	1.05	0.293	

Table S13

ANOVA table of db-RDA results (a) and variance inflation factors (VIF, b) at the European and country scale. db-RDA results were obtained by stepwise selection of variables using the AIC criterion. Only retained vairables shown here. OC: organic carbon; C/N: carbon/nitrogen ratio; Cu: bioavailable soil copper content; pH; clay = clay content, dist = soil disturbance. Df: degrees of freedom; SumOfSqs: Sum Squares; F: pseude-F value based on 9999 permutations.

**microbial group**	**data set**	**a) ANOVA**					
		**variable**	**Df**	**SumOfSqs**	**F**	**p-value**	**sig**
bacteria	EUROPE	Cu	1	3.075	22.33	0.0001	***
		CN	1	1.156	8.40	0.0001	***
		OC	1	1.366	9.92	0.0001	***
		pH	1	0.563	4.09	0.0001	***
		clay	1	1.184	8.60	0.0001	***
		Residual	391	54.096			
		R^2		0.120			
		adj. R^2		0.108			
fungi	EUROPE	Cu	1	1.911	6.33	0.0001	***
		CN	1	0.733	2.43	0.0001	***
		OC	1	1.373	4.55	0.0001	***
		pH	1	0.351	1.16	0.1378	
		clay	1	0.409	1.36	0.0202	*
		Residual	396	119.560			
		R^2		0.038			
		adj. R^2		0.026			

Appendix 5 Detailed acknowledgements

The German team would like to thank Olivia Herczynski for soil DNA extraction and is grateful for the student assistants who supported us during field work and in the lab. The contract of Brice Giffard (France) was financed in 2017 via a grant from the PromESSinG project. BG thanks Benjamin Joubard for soil sampling and DNA extraction, and Pauline Tolle (Bordeaux Sciences Agro) for setting up the management types in vineyards and coordination of the French vineyard network. The Austrian team wants to thank Lisa Cibej, Rudi Rizzoli, Sarhan Khalil and Bettina Schlossnikel for their help in data acquisition during different periods of the project. The Romanian team is grateful to Oana Paula Popa for soil DNA extraction, Sergiu Ene, Sabina Vlad, Razvan Zaharia for their help with data acquisition, and to Oana Belu, Aurora Ranca and Ion Vladoi for their collaboration and interest in the project and for granting us the access to their fields and for their collaboration in the project. The Swiss team is grateful to Andy Brown, Anne-Laure Fragnière, Franziska Keller and Mervi Laitinnen, Lara Volery and Divija Jatavallabhula as well as Nina Häner and Alix Badel for their dedication to the project and their help in data acquisition.

Supplementary references

1. Klindworth A, Pruesse E, Schweer T, Peplies J, Quast C, Horn M et al. Evaluation of general 16S ribosomal RNA gene PCR primers for classical and next-generation sequencing-based diversity studies. Nucleic Acids Res 2013; 41(1):e1.

2. Ihrmark K, Bödeker ITM, Cruz-Martinez K, Friberg H, Kubartova A, Schenck J et al. New primers to amplify the fungal ITS2 region--evaluation by 454-sequencing of artificial and natural communities. FEMS Microbiol Ecol 2012; 82(3):666–77.

3. Schoch CL, Seifert KA, Huhndorf S, Robert V, Spouge JL, Levesque CA et al. Nuclear ribosomal internal transcribed spacer (ITS) region as a universal DNA barcode marker for Fungi. Proc Natl Acad Sci U S A 2012; 109(16):6241–6.

4. FastQC: A quality control tool for high throughput sequence data.; 2010. Available from: URL: https://www.bioinformatics.babraham.ac.uk/projects/fastqc/.

5. Sickle: A sliding-window, adaptive, quality-based trimming tool for FastQ files. Version 1.33; 2011. Available from: URL: https://github.com/najoshi/sickle.

6. Schloss PD, Westcott SL, Ryabin T, Hall JR, Hartmann M, Hollister EB et al. Introducing mothur: open-source, platform-independent, community-supported software for describing and comparing microbial communities. Appl Environ Microbiol 2009; 75(23):7537–41.

7. Quast C, Pruesse E, Yilmaz P, Gerken J, Schweer T, Yarza P et al. The SILVA ribosomal RNA gene database project: improved data processing and web-based tools. Nucleic Acids Res 2013; 41(Database issue):D590-6.

8. Westcott SL, Schloss PD. OptiClust, an Improved Method for Assigning Amplicon-Based Sequence Data to Operational Taxonomic Units. mSphere 2017; 2(2).

9. Cole JR, Wang Q, Fish JA, Chai B, McGarrell DM, Sun Y et al. Ribosomal Database Project: data and tools for high throughput rRNA analysis. Nucleic Acids Res 2014; 42(Database issue):D633-42.

10. Gweon HS, Oliver A, Taylor J, Booth T, Gibbs M, Read DS et al. PIPITS: an automated pipeline for analyses of fungal internal transcribed spacer sequences from the Illumina sequencing platform. Methods in Ecology and Evolution 2015; 6(8):973–80.

11. Kõljalg U, Nilsson RH, Abarenkov K, Tedersoo L, Taylor AFS, Bahram M et al. Towards a unified paradigm for sequence-based identification of fungi. Mol Ecol 2013; 22(21):5271–7.

12. R Core Team. R: A language and envrionment for Statistical Computing; 2019. Available from: URL: https://www.R-project.org/.

13. McMurdie PJ, Holmes S. phyloseq: an R package for reproducible interactive analysis and graphics of microbiome census data. PLoS ONE 2013; 8(4):e61217.

14. Cáceres M de, Legendre P. Associations between species and groups of sites: indices and statistical inference. Ecology 2009; 90(12):3566–74.

15. Burns KN, Bokulich NA, Cantu D, Greenhut RF, Kluepfel DA, O'Geen AT et al. Vineyard soil bacterial diversity and composition revealed by 16S rRNA genes: Differentiation by vineyard management. Soil Biology and Biochemistry 2016; 103:337–48.

16. Trouvelot S, Bonneau L, Redecker D, van Tuinen D, Adrian M, Wipf D. Arbuscular mycorrhiza symbiosis in viticulture: a review. Agron. Sustain. Dev. 2015; 35(4):1449–67.

17. Zaller JG, Heigl F, Ruess L, Grabmaier A. Glyphosate herbicide affects belowground interactions between earthworms and symbiotic mycorrhizal fungi in a model ecosystem. Sci Rep 2014; 4:5634.

18. The University of Adelaide - School of Biological Sciences. Sarocladium | Mycology Online; 2021 [cited 2021 Jan 29]. Available from: URL: https://mycology.adelaide.edu.au/descriptions/hyphomycetes/sarocladium/.
